# Prediction of Psilocybin Response in Healthy Volunteers

**DOI:** 10.1371/journal.pone.0030800

**Published:** 2012-02-17

**Authors:** Erich Studerus, Alex Gamma, Michael Kometer, Franz X. Vollenweider

**Affiliations:** 1 Neuropsychopharmacology and Brain Imaging & Heffter Research Center, University Hospital of Psychiatry, Zurich, Switzerland; 2 Department of Clinical and Social Psychiatry, University Hospital of Psychiatry, Zurich, Switzerland; Catholic University of Sacred Heart of Rome, Italy

## Abstract

Responses to hallucinogenic drugs, such as psilocybin, are believed to be critically dependent on the user's personality, current mood state, drug pre-experiences, expectancies, and social and environmental variables. However, little is known about the order of importance of these variables and their effect sizes in comparison to drug dose. Hence, this study investigated the effects of 24 predictor variables, including age, sex, education, personality traits, drug pre-experience, mental state before drug intake, experimental setting, and drug dose on the acute response to psilocybin. The analysis was based on the pooled data of 23 controlled experimental studies involving 409 psilocybin administrations to 261 healthy volunteers. Multiple linear mixed effects models were fitted for each of 15 response variables. Although drug dose was clearly the most important predictor for all measured response variables, several non-pharmacological variables significantly contributed to the effects of psilocybin. Specifically, having a high score in the personality trait of Absorption, being in an emotionally excitable and active state immediately before drug intake, and having experienced few psychological problems in past weeks were most strongly associated with pleasant and mystical-type experiences, whereas high Emotional Excitability, low age, and an experimental setting involving positron emission tomography most strongly predicted unpleasant and/or anxious reactions to psilocybin. The results confirm that non-pharmacological variables play an important role in the effects of psilocybin.

## Introduction

Responses to classical hallucinogens, such as psilocybin, strongly vary between and within subjects, even when the drug dose is kept constant [1], [2]. It has therefore long been postulated that a large proportion of inter- and intraindividual differences in reactions to hallucinogens is determined by non-pharmacological variables – also often referred to as set and setting. As originally defined by Leary et al. [3], set refers to the preparation of the subject, his personality structure, and current mood state, whereas setting refers to the the physical, social, and cultural environment in which the drug is taken. Although set and setting influence the psychological effects of any psychotropic substance, including alcohol and nicotine (e.g. see [4]), the effects of hallucinogens seem to be particularly strongly determined by these conditions [1], [5]. In fact, they are not only said to be influenced by an individual subject's mental state and surroundings, but to pharmacologically amplify the impact of these non-pharmacological factors on human experience [6], [7].

Since human hallucinogen research has been dormant for almost three decades and has only come to a revival recently [8], most of what we know today about non-pharmacological predictors of hallucinogen response is based on a small number of older studies, many of which do not conform to modern methodological standards. Nevertheless, most of these studies suggest that responses to classical hallucinogens are dependent at least to some degree on the personality structure (e.g., [9]–[16]). Further influencing factors include the mood state immediately before drug intake (e.g., [14], [17]), peer-support [18], estimated emotional support [3], expectations of the subjects (e.g., [3], [14], [17]), age [17], [19], body morphology [17], size of the group in which the drug is taken [3], and drug pre-experiences [3], [17].

However, most of these studies have obtained only a limited number of potential predictors at a time. Furthermore, almost all of these studies have relied on simple correlations instead of multiple regression to investigate associations between set and setting variables and drug response. Thus, they did not adjust for potentially confounding variables and also could not reveal the order of importance of different variables. The only exception is a study by Dittrich and his colleagues [14], [20], which has used multiple regression to predict responses to *N,N*-dimethyltryptamine (DMT), nitrous oxide, and sensory deprivation from a large number of different set and setting variables. Unfortunately, the sample size of the DMT subgroup was relatively small (*n* = 45), and the study so far has only been published in book chapters.

Given these methodological problems and given that a growing number of investigators are using hallucinogens for experimental and therapeutic purposes [8], new investigations on set and setting are both timely and important. Beyond basic research, such investigations could serve the following purposes. First, they can help to improve the safety of controlled experiments using hallucinogens by providing a basis for deciding which subjects to exclude at screening and how to adjust the environment and procedures for minimizing the risk of adverse reactions. Second, they help to better standardize future experiments. For instance, treatment allocation can be improved by stratifying experimental and control groups on the most important non-pharmacological predictors and efforts in controlling confounding variables can be better directed to those that really matter. Furthermore, the most important predictors can be used for covariate adjustment in randomized controlled trials, which improves precision and power in the estimation of treatment effects [21]. Last but not least, knowledge about non-pharmacological predictors can significantly advance our understanding of the neurobiological systems involved in the actions of hallucinogens. This is because individual differences in personality, demographic characteristics, mood, etc. on the one hand, and responsiveness to hallucinogens on the other hand, could be both related to structural and functional differences in specific neurotransmitter systems. In the case of psilocybin, differences are most likely related to differential functioning and density of cortical 5-HT_2A_ receptors because this is the main site of action of classical hallucinogens [22], [23]. However, other receptors (particularly the 5-HT_1_, 5-HT_4_, 5-HT_5_, 5-HT_6_, and 5-HT_7_ receptors) and neurotransmitter systems (particularly the glutamate system), which are additionally involved in the actions of classical hallucinogens [8], [24], might also contribute to common individual differences.

Thus, to further elucidate the dependency of psilocybin response on set and setting, the present study investigates the relative importance of 24 predictor variables, including age, sex, years of education, body mass index, personality traits, drug pre-experience, mental state before drug intake, psychological distress, experimental setting, and drug dose. The analysis is based on the pooled data of 23 controlled experimental studies. Most of these have been published before as single studies. Additionally, data from eight of the 23 pooled studies (i.e., those carried out between 2000 and 2008) were used in a recent pooled analysis on acute, subacute, and long-term subjective effects of psilocybin [2] and data from 20 studies (i.e., all but the three most recent studies) were used in a recent psychometric investigation of the OAV questionnaire [25]. However, none of these studies have yet reported about the dependency of psilocybin effects on non-pharmacological predictors.

This study improves on previous predictor studies in several ways. First, the sample size (*n* = 409) is about four times as large as in the largest previous study [3]. Second, the predictor variables that we used covered a wide range of potentially important domains, and the effects of these predictors were adjusted for the most important confounders. Third, all outcome variables and most of the predictor variables were measured by validated instruments. Fourth, psilocybin was administered under highly standardized research conditions. Finally, by using modern statistical techniques, such as the bootstrap, more reliable estimates of variable importance were obtained.

## Methods

### Ethics Statement

All studies were approved by the Ethics Committee of the University Hospital of Psychiatry, Zurich, and the use of psilocybin was authorized by the Swiss Federal Office of Public Health, Department of Pharmacology and Narcotics, Berne. All subjects gave their written consent after having received detailed information about the aims of the studies, the experimental procedures involved, and the effects and possible risks of psilocybin administration. Subjects were reimbursed for their time and free to withdraw from the study at any time. To minimize potential risks of psilocybin administration, safety guidelines similar to those recommended by Johnson et al. [26] were followed in all studies.

### Pooled Studies

The sample used in the present investigation was obtained by pooling raw data from 23 experimental studies (including pilot studies) involving psilocybin administration to healthy volunteers. The studies were conducted at our research facility between 1992 and 2011 as part of a research program in which psilocybin was used as a tool for pharmacological modeling of core symptoms of schizophrenia and for studying cognitive, perceptual, and emotional processes [23], [27].

All pooled studies used placebo-controlled within-subject designs. Depending on the study, subjects received placebo and 1–4 different doses of psilocybin in 2–5 experimental sessions, each separated by at least two weeks to avoid carry-over effects. In six of the pooled studies, subjects also received a receptor blocker (i.e., buspirone, ketanserin, haloperidol, lamotrigine, and risperidone) alone and in combination with psilocybin. In the majority of the studies (*n* = 16), the order of drug administration was randomized and double-blind, but some of the earlier studies as well as most pilot studies (*n* = 7) were open-label trials.

For the present analysis, we only used data from experimental sessions in which psilocybin was administered alone and at a dose of at least 115 mg/kg po. Lower psilocybin doses were excluded because they failed to produce subjective drug effects that were statistically different from placebo [2], [28]. The pooled sample included 409 psilocybin administrations and 261 subjects. The administered psilocybin dose ranged from 115 to 315 mg/kg po (M 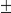 SD: 214
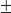
63 mg/kg).

### Subjects

Participants of all studies were recruited through advertisement from the local universities and hospital staff and carefully screened before admission to the studies. Exclusion criteria were as follows: Personal or family history of schizophrenia, major depression, bipolar, and borderline personality disorder; personal history of alcohol or illicit drug abuse; neurological disorders; and abnormal blood count, electrocardiogram, or blood pressure. Additionally, most studies excluded subjects with an Emotional Lability score in the Freiburg Personality Inventory (FPI) [29] more than two standard deviations above the mean of a normative sample. Descriptive statistics of the included subjects are presented in Table 1.

**Table 1:** Descriptive statistics of subjects (*n* = 261).

Characteristics	Values^a^	Missings
Age	27.8 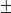 6.0	0%
Body mass index	22.2 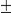 2.3	25%
Gender		0%
male	62% (161)	
female	38% (100)	
Education		0%
High school diploma	9% (23)	
University students	56% (147)	
University graduates	35% (91)	
Hallucinogen-naïve		15%
yes^b^	59% (131)	
no	41% (90)	
Daily smoker		24%
yes	30% (59)	
no	70% (139)	
THC use		16%
never	16% (35)	
rarely^c^	50% (109)	
sometimes^d^	35% (76)	
Alcohol consumption		23%
< = 60 ml per month	55% (110)	
>60 ml per month	45% (90)	
ZKPQ		
Impulsive Sensation Seeking^e^	0.4 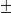 0.8	52%
Neuroticism-Anxiety^e^	−0.9 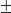 0.7	52%
Aggression-Hostility^e^	−0.6 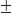 0.9	52%
Activity^e^	0.0 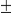 0.9	52%
Sociability^e^	−0.1 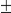 0.9	52%
TAS		
Absorption^f^	−0.8 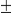 1.2	72%
SCL-90-R		
Global Severity Index^g^	−0.3 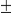 0.9	31%

*Note.* THC = Tetrahydrocannabinol; ZKPQ = Zuckerman-Kuhlman Personality Questionnaire; TAS = Tellegen Absorption Scale; SCL-90-R = Symptom Check-List-90-Revised.

a Means 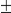 standard deviations and frequencies are shown for continuous and categorical variables, respectively. Numbers in parenthesis indicate absolute frequencies.

b Experience of a classical hallucinogen at least once in a lifetime previous to the first experimental day.

c Less than once per month.

d 1–10 times per month.

e Normed on the Bielefeld-Jena sample (*n* = 141) of Angleitner et al. [33].

f Normed on the sample of Ritz et al. [36].

g Normed on a German community sample (*n* = 1006) [40].

### Predictor Variables

Two groups of predictor variables were used: (1) Predictor variables that only varied between subjects, i.e., were constant across different drug sessions of the same individual, and (2) predictor variables that varied between and within subjects. Predictor variables of the first group were measured at screening and included age, gender, body mass index (BMI), years of education, drug use, psychological problems, and stable personality traits, whereas predictor variables of the second group were either measured at the beginning of each drug session shortly before drug administration (e.g., measures of the present mood state) or determined by the design of the experiment (e.g., drug dose, environment of the drug session, and time of assessment). Predictors of the first and second group thus describe subject (Table 1) and session characteristics (Table 2), respectively.

**Table 2:** Descriptive statistics of psilocybin sessions (*n* = 409).

Characteristics	Values^a^	Missings
Psilocybin dose (µg/kg)	214.1 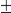 63.0	0%
Psilocybin dose (categorized)		0%
115–125 µg/kg	23% (93)	
170 µg/kg	9% (35)	
215–225 µg/kg	20% (83)	
250–270 µg/kg	38% (157)	
315 µg/kg	10% (41)	
Time of assessment^b^		0%
60–90 min	23% (96)	
110–160 min	39% (158)	
195–270 min	23% (94)	
6–10 h	13% (53)	
24 h	2% (8)	
Setting		0%
PET^c^	12% (51)	
no PET	88% (358)	

a Means 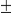 standard deviations and frequencies are shown for continuous and categorical variables, respectively. Numbers in parenthesis indicate absolute frequencies.

b Completion of OAV or 5D-ASC questionnaire after drug intake.

c Drug sessions involving positron emission tomography (PET) measurements.

#### Drug use and pre-experience with classical hallucinogens

Depending on the study, information on present and past drug use was obtained by semi-structured psychiatric interviews or by one of two different versions of investigator constructed questionnaires. The following categorical predictor variables were constructed by pooling information from all available sources: (1) “Daily smoker” is a dichotomous variable that is one if the subject smokes at least one cigarette a day and zero otherwise. (2) “Alcohol consumption” is a dichotomous variable that is one if the subject drinks more than 60 ml pure ethanol from alcoholic beverages per month and zero otherwise. (3) “THC use” is an ordered categorical variable with the three categories “never” (absolutely no experience with THC), “rarely” (less than once per month), and “sometimes” (at least once per month). THC use was represented in all regression models as two dummy coded contrast variables using an ordinal coding scheme. That is, when both variables were contained in the model, the first dummy variable represented the difference between “never” and “rarely” and the second between “rarely” and “sometimes”. (4) “Hallucinogen-naïve” is a dichotomous variable that is one if the subject has never used classical hallucinogens, such as psilocybin, LSD, and mescaline and zero otherwise. Hallucinogen-naïve is the only drug use variable that could change from one session to another within the same individual because participations on earlier experimental psilocybin sessions were also counted as lifetime hallucinogen experiences. Distributional characteristics of the four drug use variables are displayed in Table 1.

#### Zuckerman-Kuhlman Personality Questionnaire (ZKPQ) [30]

The ZKPQ contains 99 self-referent true/false statements that cover five major dimensions of personality (1) Impulsive Sensation Seeking consists of of the two facets Impulsivity (i.e., a lack of planning and tendency to act quickly on impulse without thinking) and Sensation Seeking (i.e., a general need for thrills and excitement and preference for unpredictable situations and friends). (2) Neuroticism-Anxiety describes emotional upset, worry, fearfulness, obsessive indecision, lack of self confidence, and sensitivity to criticism. (3) Aggression-Hostility reflects a readiness to express verbal aggression; rude, thoughtless or antisocial behavior; vengefulness; spitefulness; and a quick temper and impatience with others. (4) Activity consists of the two facets Need for General Activity (i.e., impatience and restlessness when there is nothing to do) and Work Activity (i.e., a preference for challenging and hard work). (5) Sociability comprises the two components Parties and Friends (i.e., a liking for big parties, interacting with many people and having many friends) and Isolation Intolerance. There is also a control scale, the Infrequency scale, that serves to eliminate subjects with possibly invalid records.

The ZKPQ is the standard instrument for the assessment of Zuckerman's alternative Five-Factor Model (FFM) of personality. In contrast to the classic FFM (the so called “Big Five”), which has been identified by lexical analyses of words describing personality, the development of the alternative FFM was guided by the assumption that basic personality traits are those with a strong biological-evolutionary basis [30]. Consequently, the primary dimensions of the ZKPQ were identified by factor analyzing scores on a variety of personality and temperament scales with known or suspected biological determinants. However, despite conceptual and methodological differences, joint factor analyses of the ZKPQ with the NEO-PI-R [31], a well established measure of the Big Five, suggest a large overlap between the two FFMs [32]. That is, the ZKPQ factors Sociability and Neuroticism-Anxiety are considered highly convergent with the Big Five factors Extroversion and Neuroticism, respectively, and the ZKPQ factors Impulsive Sensation Seeking and Aggression Hostility have shown at least moderate negative correlations with the Big Five factors Conscientiousness and Agreeableness, respectively.

The authorized German adaptation of the ZKPQ, which has shown good psychometric properties in two independent German samples [33], was used in 11 of the 23 pooled studies and completed by 125 subjects.

#### Freiburg Personality Inventory (FPI; half form B) [29]

The FPI half form B contains 105 self-referent true/false statements which – according to the authors of the instrument – measure nine primary and three secondary dimensions of personality. It was administered as part of the screening procedure in 16 of the 23 pooled studies and completed by 155 subjects. The FPI measures very similar personality traits as the ZKPQ. Thus, in order to reduce multicollinearity and to keep the number of candidate predictors low, we only used the scales of the FPI for imputing missing values of the ZKPQ, but not for predicting acute drug responses directly (see statistical analysis section for additional details). Even though the FPI was more completely assessed (40.6% missings in the FPI vs. 52.1% missings in the ZKPQ), we decided to use the ZKPQ and not the FPI for predicting psilocybin responses because the ZKPQ has undergone more extensive psychometric testing and is much more widely used internationally than the FPI half form B.

#### Tellegen Absorption Scale (TAS) [34]

The TAS is a widely used self-report questionnaire for assessing the personality trait Absorption. As measured by the TAS, Absorption refers to an individual's openness to a variety of cognitive, perceptual, and imagistic experiences as well as vivid imagery, synesthesiae, and intense involvement in aesthetics and nature. The TAS has been reported to be strongly associated with fantasy proneness, and modestly with the Big Five factor Openness to Experience and hypnotic susceptibility [35].

We used the German Version of the TAS [36] with a modified item response format (i.e., five-point Likert scale ranging from *does not apply* (0) to *does fully apply* (4) instead of the original dichotomous *true* or *false* response), which is the same version as Ott et al. [37] have used. It was administered in 4 of the 23 pooled studies and completed by 73 subjects. The internal consistency as well as the general factor saturation of the TAS in our sample were excellent (Cronbach's α = 0.95; McDonald's 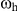 = 0.75).

#### Passive-Spontaneous Imagination (PASI)

The PASI is a subscale of the Hallucination Prediction Inventory (HPI-81; Diezi, Faeh, and Hermann, unpublished master's thesis, which was constructed to explain individual differences in experiencing visual alterations during altered states of consciousness (ASCs). It consists of 30 four-point Likert scale items measuring the frequencies of visual phenomena that spontaneously occur during hypnagogic and hypnopompic states, daydreaming, closed eyes, listening to music, thinking, and imagining (see Supplementary Table S1, for an English translation of the PASI items). The PASI was reported to have good psychometric properties in a normative sample of 442 subjects Diezi, Faeh, and Hermann, unpublished master's thesis. Furthermore, in a experiment, in which ASCs were induced by sensory deprivation (*n* = 35), the PASI was a strong predictor of visual hallucinatory phenomena as measured by the Visionary Restructuralization scale of the Aussergewöhnliche Psychische Zustände (APZ) [38] questionnaire.

The PASI was administered in 8 of the 23 pooled studies and completed by 107 subjects. The internal consistency of the PASI in our sample was excellent (Cronbach's α = 0.93), and the general factor saturation was satisfactory (McDonald's 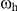 = 0.65). There was also a strong correlation of the PASI with the TAS (*r* = 0.77, *n* = 53), suggesting a large overlap between these two constructs. In order to reduce redundancy, we only used the PASI to impute missing values of the TAS, but not for predicting psilocybin responses directly. Similar to the FPI and ZKPQ, the TAS was preferred over the PASI even though it had more missing values (72% missings in the TAS vs. 59% missings in the PASI) because it is more widely used internationally and has been more extensively validated.

#### The Symptom Check-List-90-Revised (SCL-90-R; [39]; German version by [40])

The SCL-90-R is a widely used self-report inventory designed to screen for a broad range of psychological problems present in the past four weeks. Each of the 90 items is rated on a five-point Likert scale of distress ranging from *not at all* (0) to *extremely* (4). The items of the SCL-90-R are assigned to 9 different symptom dimensions: Somatization, Obsessive-Compulsive, Interpersonal Sensitivity, Depression, Anxiety, Anger-Hostility, Phobic Anxiety, Paranoid Ideation, and Psychoticism. However, because these nine dimensions are not supported by most exploratory and confirmatory factor analyses and because many studies have pointed to the presence of a strong general factor [41], only the Global Severity Index (GSI), which is the total score of all SCL-90-R items, was used as a predictor variable.

The SCL-90-R was administered as part of the screening procedures in 14 of the 23 pooled studies and completed by 179 subjects. The internal consistency of the GSI in the pooled sample was excellent (Cronbach's α = 0.94), and the general factor saturation was satisfactory (McDonald's 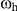 = 0.62).

#### Adjective Word Lists (“Eigenschaftswörterliste”; EWL-60-S and EWL-K)

Two different versions of the Adjective Word List were used to assess the current mental state shortly before drug administration. The older version EWL-K [42] was used in studies before the year 2000 (*n* = 7), whereas the newer version EWL-60-S [43] was used in later studies (*n* = 5). Both questionnaires contain a list of adjectives which must be rated on how well they describe the current mental state. The EWL-K contains 123 adjectives and a dichotomous *true* or *false* response format, whereas the EWL-60-S contains 60 adjectives and a four-point response format ranging from *not at all* (0) to *strongly* (3). In both questionnaires, items are grouped into six main scales: Performance-Related Activity, General Inactivation, Extroversion-Introversion, General Well-Being, Emotional Excitability, and Anxiety-Depressiveness. We combined these scales across questionnaire versions by using only those items that are contained in both questionnaire versions (see Supplementary Table S2 for a list of the overlapping items in each scale). To adjust for the different item response format, each EWL scale was z-transformed within each questionnaire version. By combining EWL-K and EWL-60-S, measures of the mental state before drug intake were available from 185 of the 409 drug sessions.

### Response Variables

#### Altered States of Consciousness Rating Scales OAV and 5D-ASC

In each experimental session, subjects were asked to rate drug induced alterations of consciousness by either the OAV questionnaire [44] or its extended version 5D-ASC [45]. The OAV was used in studies conducted before the year 2000 (*n* = 10), whereas the 5D-ASC was used in all later studies (*n* = 13). In all studies, questionnaires were administered during the acute or post-acute effects of the drug and subjects were asked to rate their experiences from the moment of drug intake to the time of assessment. If the questionnaires were completed more than once during an experimental session, only data from the measuring time points yielding the highest mean total score were used. The frequencies of different assessment times in the pooled sample are shown in Table 2. Because the time of assessment could have affected subjective drug effects ratings, time (defined as the logarithm of minutes after drug intake) was included as covariate in all statistical analyses.

There are 66 visual analogue items that occur in both the OAV and 5D-ASC and that can be used to assess three primary and one global dimension of ASCs. The three primary dimensions are called Oceanic Boundlessness (OBN), Dread of Ego Dissolution (DED), and Visionary Restructuralization (VRS), and the global dimension is called Altered States of Consciousness (G-ASC). The OBN dimension describes highly enjoyable and positively valued experiences of ASCs, such as deeply felt positive mood, experiences of unity, transcendence of time and space, spiritual experiences, and sense of intuitive understanding. Because many of the OBN items have been directly formulated on the basis of six of the nine categories of mystical experiences proposed by Stace [46], high scores on the OBN scale indicate a state similar to mystical experiences as described in the scientific literature on the psychology of religion. The DED dimension measures experiences of cognitive impairment, loss of self-control, feelings of disintegration or separation from oneself and the world, and anxiety or panic. High scores on the DED scale therefore indicate a very unpleasant state similar to so called “bad trips” described by drug users. The VRS dimension assesses elementary and complex visual pseudo-hallucinations, audio-visual synesthesiae, increased production of vivid imagery from memory or fantasy, as well as changes in the meaning of percepts. Finally, the secondary scale G-ASC is the total score of all 66 OAV items and thus can be considered as a general measure of consciousness alteration.

The OBN, DED, VRS, and G-ASC dimensions have been hypothesized to be fundamental dimensions of ASCs that are factorially invariant across ASCs induction methods [38]. However, a recent psychometric investigation of the OAV [25] has only partially confirmed this hypothesis. Specifically, it has been found that the VRS factor contains several items that load more strongly on the OBN factor and that the VRS factor could be merged with the OBN factor on a high level of construct hierarchy. Furthermore, all original OAV factors were demonstrated to be multidimensional. Studerus et al. [25] therefore constructed and validated eleven new lower order factors that are more homogeneous than the original factors and that can be used to describe more specific aspects of ASCs (see [25] for descriptions of these scales). Nevertheless, because the original factors have shown relatively strong general factor saturations, they still can be advantageous for capturing complex criteria. To this end and in order to compare our results with earlier studies, we decided to use both the original and the recently constructed subscales as dependent variables.

### Statistical Analysis

To ensure the validity of the assumptions of linear mixed effects models (i.e., Gaussian distribution of random effects and within-subjects errors, homoscedasticity, and linearity), an extended method of Box-Cox transformation [47] was applied to all response variables. The negative inverse of the square root was found to be appropriate for the Anxiety factor, and a natural logarithm transformation worked best for the DED, Spiritual Experience, Insightfulness, Disembodiment, and Audio-Visual-Synesthesiae factors. All other response variables were transformed by taking the square root. Predictor variables were not transformed because partial residual plots indicated that linearity assumptions were already reasonably well satisfied after transforming the response variables.

As depicted in Supplementary Figure S1, several predictor variables contained considerable proportions of missing values. Because the missing data mostly resulted from different study designs among the pooled studies, the missing data mechanism can be assumed to be “missing at random” (MAR) or “missing completely at random” (MCAR) [48]. To minimize potential bias and loss of information arising form missing data, we used a statistical technique called multiple imputation (MI) [49]. MI is regarded as the method of choice for handling complex incomplete data problems because it yields unbiased parameter estimates and standard errors under an MAR or MCAR missing data mechanism and maximizes statistical power by using all available information [48]. We imputed missing values by the Multivariate Imputation by Chained Equations (MICE) software [50], which is freely available as an add-on package to R [51]. The MICE-package uses fully conditional specification as imputation method, which means that imputation models can be flexibly specified on a variable-by-variable basis. We used predictive-mean-matching, proportional odds models, and logistic regressions to impute continuous, ordered categorical, and binary variables, respectively. The scales of the the FPI and PASI questionnaires were included in the MI procedure as auxiliary variables to improve the imputation of the ZKPQ and TAS scales, respectively. For each variable, the set of predictors was restricted to those that correlated with at least 0.15 with the variable to be imputed. This resulted in a series of imputation models that contained the best 9–29 predictors of each target variable. Due to the relatively large fraction of missing information in some variables, we generated 20 multiply imputed data sets, which is a larger number than what the literature historically recommends [48]. Recent simulation studies (e.g., [52]) show that this has a very beneficial impact on statistical power, especially when the fraction of missing information is as high as in the present study. Convergence of the Gibbs sampling algorithm and the quality of imputed values were assessed in accordance with recommendations of Buuren et al. [50].

To ensure that no severe multicollinearity existed between predictor variables, variance inflation factors (VIFs) were computed for each predictor variable within in each of the imputed data sets. Because no VIF was larger than 3, we did not exclude any predictor variable due to multicollinearity.

Because some subjects participated in more than one psilocybin study and because some studies involved multiple psilocybin sessions, our pooled data set contains non-independent observations. To account for this non-independency, we used linear mixed models in which the intercepts were allowed to vary per subject. We also considered more complex mixed effects models with varying slopes for the drug dose effects and varying intercepts by study. However, model comparisons by the Akaike's information criterion (AIC) in the full models suggested that the varying intercept per subject model was sufficient to account for the clustering in our data.

In order to directly compare regression coefficients of binary and continuous predictors, continuous predictor variables were rescaled within each imputed data set by dividing them by two times their standard deviations. Because binary variables – except when highly skewed – have a standard deviation of roughly 0.5, our rescaling procedure resulted in regression coefficients that reflected the change of the dependent variable for a two standard deviation change in both binary and continuous predictors (see also [53]). Outcome variables were z-transformed within each imputed data set such that regression coefficients were also comparable across models with different outcomes.

Regression models that contain too many unimportant predictor variables can result in loss of precision in the estimation of regression coefficients and the predictions of new responses [54]. On the other hand, selecting variables by data-dependent methods (e.g., stepwise approaches) may result in overly optimistic estimates of predictive ability and model fit and unstable sets of predictor variables, especially in small data sets [21]. To reduce these risks, we built our models by combining backward elimination with a two step bootstrap approach. A major advantage of this approach is that it also solves the problem of variable selection under multiple imputation (e.g., [55]).

In the first step, 200 bootstrap samples were taken from each of the 20 imputed data sets. The bootstrap samples were obtained by drawing from individual cases (i.e., psilocybin sessions) with replacement and were of equal sample size as the original sample. Within each bootstrap sample, parsimonious models were searched for by applying backward elimination. That is, starting from the full models, predictors were dropped in a stepwise fashion until no predictor was left with a Wald test *p*-value larger than 0.157. This significance level corresponds to selecting predictors with 1 *df* based on the AIC [21]. The random intercept for the subjects was always included in the models and only fixed effects were considered for elimination. Predictors were ranked according to their inclusion frequencies in the final models. Those predictors that were selected in at least 50% of the 20
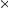
200 bootstrap samples were considered important and further analyzed in a second modeling step. Thus, the first step primarily served to reduce model space with a minimal risk of eliminating important predictors (see also [56]).

The second modeling step was very similar to the first step. Again, 200 bootstrap samples were taken within each imputed data set and backward elimination was applied with a stopping rule of *p* = 0.157. However, this time we began from full models that included only those predictors that were selected in the first step. The model that was selected most often across all bootstrap samples was considered the most stable model and further explored for relevant interactions. The two step bootstrap procedure was repeated for each of the 15 response variables.

In each of the resulting 15 final models, the multivariate associations between the repeatedly measured outcomes and the fixed effects in the models were estimated by the *R^2^* statistic proposed by Edwards et al. [57]. To obtain reliable standard errors, confidence intervals, and associated *p*-values of the fixed effects parameters, Markov Chain Monte Carlo (MCMC) sampling was used. MCMC sampling is a modern alternative to the conventional significance test of fixed effects in mixed effects models based on *t* or *F* statistics, which is unreliable due to the lack of a clear definition of the degrees of freedom [58]. For each final model and each imputed data set, random draws from the posterior distributions of the parameters were taken and then mixed across data sets. The mixed draws approximate the posterior distribution of the pooled parameters and thus can be used for inference after multiple imputation [59]. A simulation study by Zhou et al. [60] has shown that this approach leads to better results than the conventional application of Rubins's rules [49], especially when the number of imputed data sets is large.

## Results

The selection frequencies of the predictor variables and models as a result of the bootstrap selection procedures are provided in Supplementary Table S3. Drug dose was the only predictor variable that reached a selection frequency of 100% and it did so with all 15 response variables. The number of predictor variables that were selected in more than 50% of the bootstrap samples ranged from 5 for the dependent variable DED to 20 for the dependent variable Audio-Visual Synesthesiae. BMI and daily smoker were the only variables that never reached a selection frequency of 50%. In general, the number of predictor variables reaching the cutoff of 50% was considerably lower for the original OAV scales than for OAV subscales (6–7 vs. 16–24). The lower number of selected variables in the first step also led to more stable models in the second step. Whereas the most frequently selected model of the dependent variable OBN was selected in 32.1% of the cases, the most frequently selected model of the dependent variable Anxiety was selected only in 0.3% of the cases.

Because drug dose was clearly the most important predictor, first order interactions between drug dose and all other predictors were explored within the most frequently selected models. Only 12 of the 15
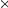
23 tested interactions were significant at *p*<0.05 and only one interaction, namely, PET
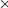
Drug Dose predicting Anxiety, reached significance at p<0.01. The interaction indicated that Anxiety increased with increasing drug dose in the non-PET condition, but decreased with increasing drug dose in the PET condition. However, it should be noted that the variability of the drug dose variable within the PET condition was very small. Specifically, only 215 and 250 mg/kg doses of psilocybin were administered in experiments involving PET measurements. Thus, the significance of this interaction was considered rather questionable and not included in the final models.

The variances explained in the full and simplified final models of all 15 outcome variables are presented in Table 3. As can be seen from the table, the variable selection procedure only slightly reduced explained variances, suggesting that the most important predictors were retained in the models, and the excluded variables were mostly noise variables. The variance explained in the simplified models was highest for the OAV total scale (*R^2^* = 0.31) and lowest for Disembodiment (*R^2^* = 0.163). In general, the main scales tended to have higher explained variances than the subscales even though they were explained by a lower number of predictors.

**Table 3:** Variance explained (Edward's *R^2^*) in the full and simplified models.

Outcome	Full	Simplified
	models	models
Main scales		
Altered state of consciousness	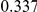	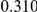
Oceanic boundlessness	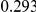	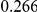
Dread of ego dissolution	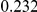	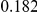
Visionary restructuralization	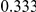	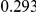
Subscales		
Experience of unity	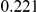	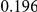
Spiritual experience	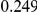	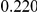
Blissful state	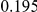	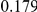
Insightfulness	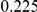	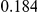
Disembodiment	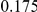	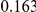
Impaired control and cognition	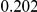	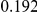
Anxiety	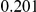	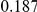
Complex imagery	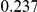	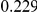
Elementary imagery	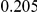	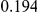
Audio visual synesthesiae	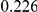	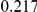
Changed meaning of percepts	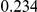	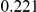

**Figure 1:**
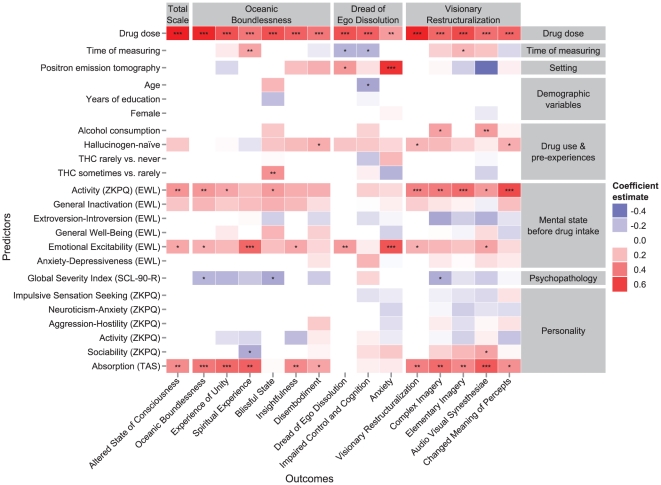
Regression coefficients of the final models pooled across 20 imputed data sets. The effects are adjusted for the influences of all other variables in the models. One, two, and three asterisks represent *p*-values 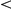
0.05, 0.01, and 0.001, respectively.

The size and statistical significance of the regression coefficients of the most stable models, as estimated by the MCMC sampling method, are shown in Figure 1. Standard errors and highest posterior density 95% credibility intervals are additionally provided in Supplementary Table S3. Overall, drug dose had the strongest effect on psilocybin response. It was significantly associated with all outcome variables and had the highest effect size in all models, except in the models predicting Spiritual Experience, Anxiety, and Changed Meaning of Percepts. The time of assessment was positively associated with Spiritual Experience and Elementary Imagery and negatively associated with DED and Impaired Control and Cognition, indicating that disturbances in control and cognition were less often reported when asked about later in the session. In experimental sessions involving PET measurements, participants reported much higher levels of Anxiety. In fact, of all 24 analyzed predictor variables, PET was the strongest predictor of Anxiety, and its effect size was more than twice as high as the one of drug dose. Compared to younger subjects, older subjects reported less Impaired Control and Cognition and also showed a trend for more Blissful State (*p* = 0.059). Years of education, gender, and BMI were not significantly associated with any response variable.

Drug use and pre-experiences with hallucinogenic drugs only moderately affected psilocybin responses. Although hallucinogen-nave subjects tended to report stronger effects in most outcome variables, statistical significance was only reached for Disembodiment, VRS, and Changed Meaning of Percepts. Psilocybin responses did not differ between subjects who never consumed THC and those who rarely consumed THC. However, subjects who sometimes smoked cannabis (more than once per month) reported significantly more Blissful State than subjects who rarely consumed cannabis (less than once per month) and also showed a trend for less Anxiety (*p* = 0.07). There were also statistically significant positive associations between alcohol consumption and experience of Audio-Visual Synesthesiae and Complex Imagery.

The mental state immediately before drug intake had a relatively strong influence on several outcome variables. Specifically, Performance-Related Activity, which was measured by the adjectives go-getting, avid, active, and energetic, had a major influence on the overall consciousness alteration (G-ASC) and on several experiences covered by the OBN and VRS dimensions. Emotional Excitability was strongly positively associated with Spiritual Experience and Anxiety and moderately with all OAV main scales, as well as Insightfulness and Audio-Visual Synesthesiae. Anxiety-Depressiveness before drug intake did not lead to significantly more unpleasant experiences during the sessions.

The GSI scale of the SCL-90-R was negatively associated with OBN, Blissful State, and Complex Imagery, indicating that subjects who experienced more psychological problems previous to the experiments reported less pronounced effects with these scales. Except for Absorption, which strongly predicted several experiences measured by the OBN and VRS dimensions, most personality traits did not have a major influence on psilocybin responses. Of the personality traits constituting Zuckerman's alternative five-factor model, only Sociability was significantly associated with any outcome variable. Specifically, subjects who were more sociable (i.e., outgoing and extroverted) reported less Spiritual Experience and more Audio-Visual Synesthesiae.

The fractions of missing information (FMI) and the relative increases in variance due to missingness (RIV), which quantify the missing data's influence on the sampling variance of the parameter estimates, are shown in Supplementary Table S3. For all imputed variables, FMI values were lower than their missing data rates, which indicates that the variables in the imputation model were predictive of the missing values. Because some of the information loss was mitigated by borrowing information from correlated variables, increases in sampling errors of the regression coefficients were not completely commensurate with overall reductions in sample sizes. Not surprisingly, the RIV was largest for Absorption (0.94 on average), which was also the variable with the highest missing data rate. This indicates that the confidence interval of the regression coefficient for Absorption was on average about 0.94 times larger than it would have been if this scale had no missing values.

## Discussion

The present study sought to predict acute responses to psilocybin when administered in a controlled scientific setting to healthy volunteers. The relative importance of 24 predictor variables from a wide range of domains were investigated.

Drug dose was clearly the most important predictor of psilocybin response. It was the only predictor that was always retained in automatic variable selection and its effect size was largest in 12 of the 15 final prediction models. Furthermore, its effect on general consciousness alteration, as measured by the OAV total scale, was more than twice as high as that of other predictors. The personality trait of Absorption was found to be the second most important predictor of psilocybin response. It was highly positively associated with the overall consciousness alteration and strongly predicted mystical-type experiences and visual effects induced by psilocybin. Further variables that were found to be important for predicting psilocybin response were Performance-Related Activity, Emotional Excitability, psychological distress as measured by the GSI, pre-experience with classical hallucinogens, frequencies of THC and alcohol consumption, Sociability, time of assessment, and setting (PET vs. no PET measurement). Being in an emotionally excitable and active state immediately before drug intake, having experienced few psychological problems in the past weeks, no previous experience with classical hallucinogens, and moderate THC and alcohol consumption increased the intensity of pleasurable effects and/or visual alterations, whereas settings involving PET measurements, Emotional Excitability, and low age contributed to the experience of unpleasant and/or anxious reactions.

The finding that Absorption was amongst the most important predictors of psilocybin-induced ASCs is consistent with a large number of studies showing that Absorption is associated with differential responsivity to various ASC induction procedures, including hypnosis, meditation, marijuana intoxication, and electromyograph biofeedback [61], [62]. Absorption has also been reported to be positively associated with the occurrence of synesthesiae after the ingestion of ayahuasca [16], a hallucinogen with similar modes of action as psilocybin. This is in agreement with our results, which showed that, of all 15 response variables, Absorption most strongly predicted Audio-Visual Synesthesiae. A recent study by Ott et al. [37] suggests that inter-individual differences in Absorption and responsiveness to hallucinogenic drugs could be both related to the binding potential of the 5-HT_2A_ receptor, which is the main site of action of serotonergic hallucinogens, such as psilocybin [8]. Although Ott et al. [37] have demonstrated a significant association between T102C polymorphism affecting the binding potential of the 5-HT_2A_ receptor and the TAS scale, they did not assess the association between TAS and responsivity to serotonergic hallucinogens. The present study is filling this gap, as it is, to our knowledge, the first study predicting the effects of a classical hallucinogen by Absorption in a large sample of subjects.

Apart from a strong influence of Absorption and a relatively minor influence of the ZKPQ factor Sociability, which is highly convergent with the Big-Five factor Extroversion [32], personality traits only marginally contributed to the prediction of psilocybin responses. This is rather surprising because personality traits have been postulated by many authors to be among the most important determinants of hallucinogen response (e.g., [11], [12]). It is also worth noting that we did not detect any statistically significant relationship between Neuroticism-Anxiety and negative reactions to psilocybin. This finding contradicts several earlier, smaller scaled studies [13]–[15], which have found moderate to strong correlations between Neuroticism and anxious reactions to classical hallucinogens and which have led to our policy of excluding subjects with very high Neuroticism scores (i.e., more than two SD above the mean) at screening. Although the exclusion of highly neurotic subjects could have distorted our sample and thus reduced the predictive ability of Neuroticism, it should be noted that the chosen cutoff affects only the highest 2.3% of the normal distribution and that there was still substantial variability of Neuroticism in our sample. Nevertheless, as has been shown in Table 1, both mean and variance of Neuroticism in our sample where somewhat reduced compared to normative data. Hence, we cannot rule out the possibility that Neuroticism increases the risk of adverse reactions in the highest tail of the distribution. A positive relationship between Neuroticism and the effects of classical hallucinogens would also be biologically plausible because a recent PET study has demonstrated a positive correlation between Neuroticism and frontolimbic 5-HT_2A_ receptor binding [63]. Thus, excluding highly neurotic subjects at screening might still be a sensible approach for increasing the safety of controlled experiments involving hallucinogen administration.

In contrast to personality factors, current mood state and psychological distress in the past four weeks before drug intake were generally more important for predicting psilocybin response in this study. This is in agreement with the existing literature. For instance, Metzner et al. [17] have found that the best predictor for mood during the psilocybin session was mood before the session, and Dittrich [14] reported that Emotional Lability, a factor that was predominantly measured by state variables, most strongly increased the likelihood of experiencing DED after DMT administration. Interestingly, we have found that Emotional Excitability shortly before drug intake predicted anxious reactions to psilocybin much better than Anxiety-Depressiveness. However, this could also be due to statistical reasons. Whereas Emotional Excitability was measured by 11 items, Anxiety-Depressiveness was measured by only 4 items that additionally also had relatively high item difficulties. Consequently, the variability – and possibly also the reliability – of Anxiety-Depressiveness was substantially lower than that of Emotional Excitability. It should also be noted that these two factors were relatively highly correlated in our sample (*r* = 0.5), which frequently might have led to the inclusion of only one of these two variables in automatic model selection. The finding that Performance-Related Activity was amongst the most important predictors of experiences described by the OBN and VRS dimensions has, to our knowledge, not been described in the literature before. One possible explanation is that the items assessing Performance-Related Activity (i.e., go-getting, avid, active, and energetic) not only captured variance associated with fitness and energy, but also variance with positive mood and general optimism. Correlations with EWL subscales (not reported) are in support of this hypothesis because they reveal that Performance-Related Activity is most strongly associated with the EWL subscale Heightened Mood (*r* = 0.47).

The finding that the PET environment was strongly associated with anxious reactions could be partially explained by the perceived atmosphere at the PET center. Whereas non-PET experiments were mostly conducted in laboratory rooms that were furnished in an aesthetically pleasing way, the environment at the PET center was much more clinical and “antiseptic” (i.e., lots of technical equipment, white walls, personnel in white lab coats). Our results are therefore in support of current safety guidelines [26], which recommend avoiding “cold” and overly clinical environments in human hallucinogen research in order to reduce the risk of anxious reactions. Although we have found increased Anxiety in PET experiments, that does not mean that psilocybin experiments involving PET measurements are unsafe. The percentage of strong anxious reactions in the PET experiments was still relatively low, and all of them could be successfully managed by providing interpersonal support. Furthermore, there are other factors that might have contributed to the increased Anxiety in the PET environment. For instance, in contrast to non-PET experiments, subjects could have their eyes closed while lying in the scanner and they were less distracted by performing tasks. Thus, they could concentrate more on the experience, which in turn might have increased the confrontation with inner fears.

Our results indicate that, in contrast to MDMA [64], the effects of psilocybin were not moderated by gender. This is consistent not only with earlier studies investigating the subjective effects of classical hallucinogens in humans [3], [19], but also with neuroimaging studies, which have found no gender differences in 5-HT_2A_ receptor binding in cortical regions [63], [65]. The only demographic variable that was statistically significantly associated with any psilocybin response in this study was age. Specifically, older subjects reported less Impaired Control and Cognition and tended to experience more Blissful State compared to younger subjects. These associations are similar to those observed by Hyde [19] and Metzner et al. [17] and could be explained by an increased experience with managing occurrent negative emotions in older people [66]. It is also consistent with the fact that 5-HT_2A_ receptors densities decrease with increasing age (e.g., [65]).

The finding that hallucinogen-naïve subjects reported slightly more VRS, Disembodiment, and Changed Meaning of Percepts is consistent with the study of Metzner et al. [17], which found that previous experience with classical hallucinogens was negatively associated with the number of somatic symptoms and visual alterations induced by psilocybin. However, our results disagree with those of Dittrich [14], who found that familiarity with drug-induced ASCs was not predictive of any acute effects of DMT, as measured by the OAV questionnaire. One possible explanation of this discrepancy might be that the predictor variable “familiarity with drug-induced ASCs” in the study of Dittrich not only included pre-experience with classical hallucinogens, but also other psychotropic substances.

Although subjects were asked to rate their experiences in retrospect and mostly during or after the peak effects of the drug, Impaired Control and Cognition induced by psilocybin was rated as less intense and Spiritual Experience and Elementary Imagery were rated as more intense when questionnaires were completed later in the sessions compared to earlier in the sessions. These findings are in agreement with a study of Linton et al. [67], which found that subjects tended to forget ego-alien and threatening aspects of an LSD experience more often than those dealing with affects or changes in the perceived meaning of events. Although it is tempting to explain these associations by the well-known phenomenon of “motivated forgetting” [68], they could also have resulted from differential time courses of psilocybin effects (e.g., see [2]).

### Limitations

The present study has several limitations. First of all, the design of our study does not allow causal interpretations of predictor effects. Although we analyzed data from experimental studies, the only variable that was systematically manipulated was drug dose and only within, not between, studies. Hence, with the exception of drug dose, associations between predictors and outcomes are purely observational.

Although several statistically significant relationships between non-pharmacological predictors and outcome variables were detected, there were still relatively large proportions of unexplained variances in the outcome variables. For instance, more than 80% of the variance of the outcome variable Anxiety was left unexplained, suggesting that there is considerable unpredictability in anxious reactions to psilocybin – even under highly standardized conditions.

Generalizations of our results are hindered by the composition of our sample and the circumstances in which psilocybin was administered. For instance, our subjects were relatively young, highly educated, and high-functioning. They had more pre-experiences with classical hallucinogens and cannabis than their corresponding age group in the general population and also showed low Neuroticism-Anxiety scores (i.e., almost one SD below the mean of a normative sample). The distortions in our sample most likely resulted from our recruitment method, which is prone to self-selection bias (see also [2]). The specific composition of our sample and the fact that psilocybin was administered in a carefully monitored research environment might have reduced the occurrence of unpleasant reactions (i.e., so called bad or horror trips). This in turn might have lowered our ability to detect risk factors for unpleasant reactions.

The individual studies that were pooled for the present analysis were not specifically designed to investigate predictors of psilocybin response. Consequently, the predictor variables analyzed herein are not necessarily those that – according to the literature – would be most promising to investigate. Although the studied predictors cover the most important domains, some of them are clearly underrepresented. For instance, the influence of the setting was only covered by the PET vs. no PET variable. Furthermore, expectancies of the subjects, which are well known to influence the effects of most psychoactive drugs, including alcohol and nicotine [69], could not be studied because no such variables were obtained.

Because the majority of the pooled studies used double-blind placebo-controlled designs, one might argue that we could have controlled for expectancy effects by including the response to placebo as a covariate into the analyses. Unfortunately, the effects of psilocybin were so strong that most subjects could easily differentiate them from placebo. Moreover, because the items of the OAV questionnaire are visual analogue, anchored *no, not more than usual* on the left and *yes, much more than usual* on the right, most subjects placed marks at the left end of the scale for all items once they were convinced that they had received placebo. Consequently, mean and variances of the OAV scales were essentially zero under placebo, which severely limited the usefulness of these scales as covariates. While some investigators have used an active placebo to increase the success of the double blind in experiments involving hallucinogens (e.g., [70]), an even better approach for separating pharmacological effects from the cognitive expectations of receiving the drug and its effect might be the so called balanced-placebo design (BPD) [71]. The BPD is a 2
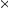
2 factorial design that crosses the administered substance (drug vs. placebo) with an instructional set manipulation (subjects are told they receive the active drug vs. subjects are told they receive placebo). To our knowledge, the BPD has not yet been used in experiments with classical hallucinogens, but a recent study has demonstrated its feasibility with marijuana [72]. It is therefore conceivable, that the BPD could also foster our understanding of expectancy effects in responses to classical hallucinogens.

Another limitation of the present investigation is that responses to psilocybin, as measured by the OAV, could be confounded by individual differences in the interpretation of the item anchors at the right end of the visual analogue scale. Specifically, the anchor *yes, much more than usual* could have had different meanings depending on whether the subject has experienced profound ASCs before. Future studies should therefore validate our results by also using behavioral measures and/or external raters for assessing psilocybin response.

In the present study, we have only predicted single aspects of ASCs. Another approach, taken by Barr et al. [12], is to predict patterns of psilocybin responses. This could be accomplished by cluster analyzing individual responses using Pearson correlations as a proximity measure. The response clusters could than be predicted by multinomial regression models. Because psilocybin, especially with higher doses, sometimes can elicit responses that are not only quantitatively but also qualitatively different [1], it is possible that a categorical approach would be better suited to detect determinants of profound ASCs, such as mystical-type experiences or so called “horror trips”. The main reason why we did not follow such an approach in this investigation is that these experiences only occurred in a small proportion of our subjects (cf. [2]). Hence, even with our large sample, the event-per-variable ratio and statistical power were considered too low for such an analysis.

A few further statistical issues are worth noting. Although we used a two-step bootstrap procedure to protect against the dangers of data-driven model selection, the stability of some prediction models were relatively low. For instance, the most frequently selected model of the outcome variable Anxiety was selected in only 0.3% of the cases, and there were many competing models that were only slightly less frequently selected. Thus, there was considerable uncertainty in some of the final models, which could have introduced bias in the estimation of regression coefficients and confidence intervals [21]. The natural remedy for this problem would have been to base inference on a set of competing models using model averaging and the selection frequencies as model weights [56]. However, because our analysis was already complicated by the fact that we had used mixed effects models in combination with multiple imputation, we did not want to introduce additional complexity into the analysis and therefore abstained from performing frequentist model averaging.

The relatively large proportion of imputed values in some predictor variables (up to 70%) might cause distrust in our results. However, it should be noted that the applied MI procedure completely protects against false inference, as long as the missing data mechanism is correctly modeled and the MAR or MCAR assumptions are met [48]. Even a predictor with 90% missing values could still be estimated with MI, albeit with relatively large uncertainty [21]. There are several reasons why we believe that the high missing data rate is not a major problem in the present investigation. First, the MAR assumption is highly plausible because missing data almost exclusively resulted from different study designs among the pooled studies. Second, the number of imputed data sets was relatively high, which is recommended with large proportions of missingness [48]. Third, even for the predictor with the highest missing data rate (i.e., Absorption), the loss of statistical power induced by missingness was moderate and did not inhibit the detection of statistically significant associations.

### Conclusions

Although drug dose was clearly the most important determinant of psilocybin response, the results of this study confirm that a substantial proportion of the intra- and interindividual differences in acute responses to psilocybin is related to differences in set and setting. The results suggest that important predictors of psilocybin response can be found in a wide range of different domains, including personality, current mood, psychopathology, drug pre-experience, demography, and environment.

## Supporting Information

Figure S1 Left: Barplot of the proportions of missing values in each questionnaire. Right: All existing combinations of of missing (red) and nonmissing (blue) values in the observations.
(PDF)

Table S1 English translation of the Passive-Spontaneous Imagination Questionnaire.
(PDF)

Table S2 Overlapping items of the EWL-K and EWL-60-S questionnaires.
(PDF)

Table S3 Selection frequencies of variables at Step 1 and models at Step 2 as a result of the bootstrap model selection process (top) and pooled parameter estimates of the most frequently selected models (bottom).
(PDF)
