# Supplementary material for: Prediction of Psilocybin Response in Healthy Volunteers
Source: PLoS One. 2012 Feb 17;7(2):e30800. doi: 10.1371/journal.pone.0030800 (PMC3281871; doi:10.1371/journal.pone.0030800)
Supplement: Figure S1 — Left: Barplot of the proportions of missing values in each questionnaire. Right: All existing combinations of of missing (red) and nonmissing (blue) values in the observations. (PDF) [file pone.0030800.s001.pdf]

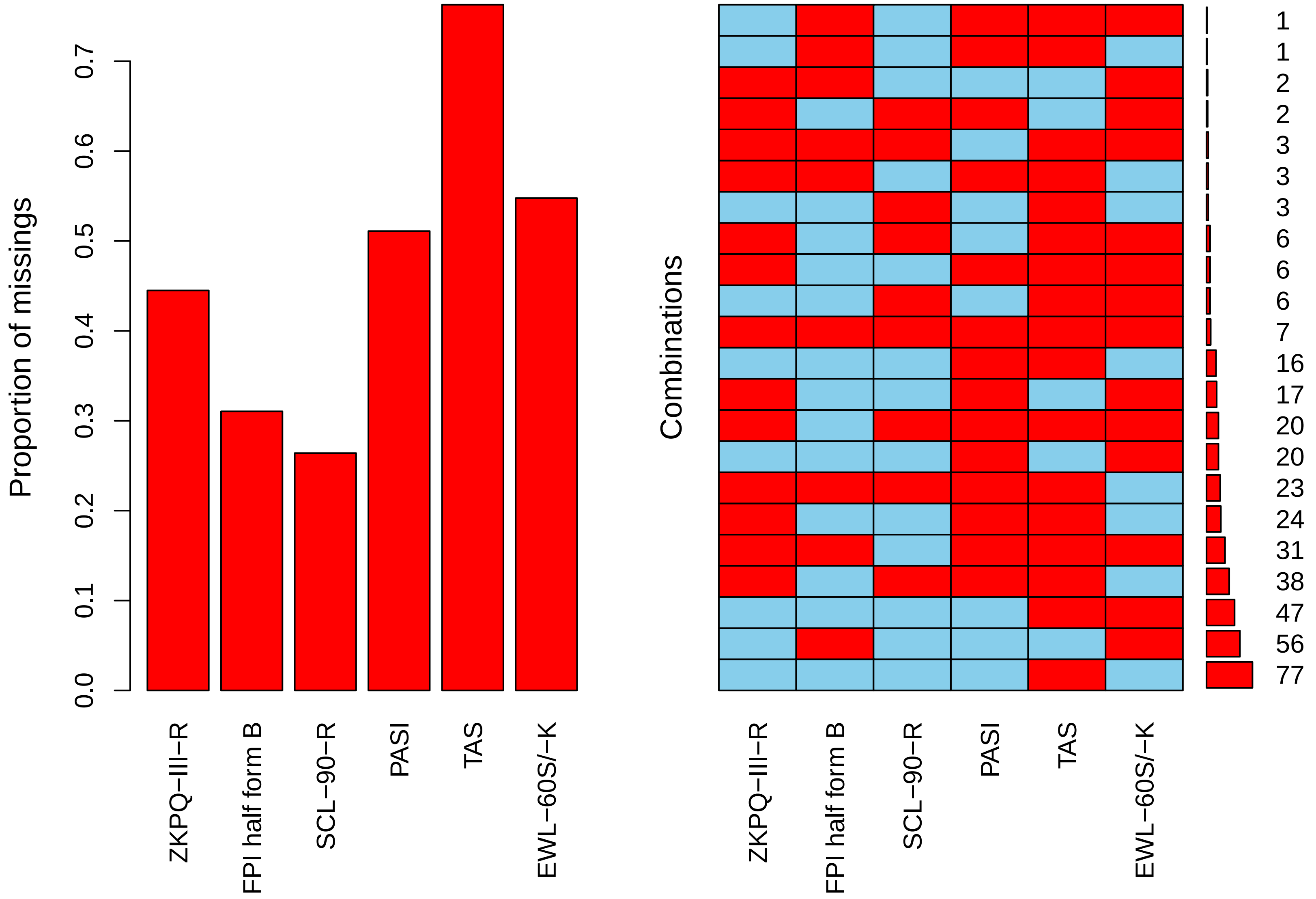

Supplementary Figure 1. Left: Barplot of the proportions of missing values in each questionnaire. Right: All existing combinations of of missing (red) and non−missing (blue) values in the observations.
