# Supplementary material for: Prediction of Psilocybin Response in Healthy Volunteers
Source: PLoS One. 2012 Feb 17;7(2):e30800. doi: 10.1371/journal.pone.0030800 (PMC3281871; doi:10.1371/journal.pone.0030800)
Supplement: Table S1 — English translation of the Passive-Spontaneous Imagination Questionnaire. (PDF) [file pone.0030800.s002.pdf]

Supplementary Table S1. English translation of the Passive-Spontaneous Imagination Questionnaire.

1.	When waking up I am able to see lively images, while my eyes are still closed.
2.	When listening to music intensely, it can occur that I see images.
3.	It occasionally occurs that my day-dreams distract me from important affairs.
4.	I only need to close my eyes and I already see colors.
5.	It can occur that my day-dreams distract me from my day to day tasks.
6.	When falling asleep, I see colors.
7.	It has occurred that I have imagined something so intensely, that it seemed to become reality.
8.	When looking through holiday photographs, I imagine entire scenes from my holiday memories with vivid precision.
9.	When something unpleasant enters my mind, I see images.
10.	It has occurred, that no matter how hard I tried I could not remember the name of a relative or close friend, although I could see the image of his/her face clearly in front of me.
11.	It sometimes happens that on long car or train journeys, I begin to visualize imagines so intensely that my perception of the outside world begins to fade.
12.	When falling asleep, I see image.
13.	It occurs that by looking at an object intensely I get the feeling that it starts to change right in front of me.
14.	It can occur that when I close my eyes I see whole scenes.
15.	It has happened that for a fraction of a second I believed to have seen a person in front of me, who could never have been there in reality.
16.	I have seen creatures or beings chase each other across the sky on cloudy days.
17.	My day-dreams are so vivid and clear, that they feel as though they are real.
18.	While I work, images from my own life seem to flow past my mind’s eye.
19.	It has occurs that in the dark I can get agitated because I get the impression that something is moving.
20.	When falling asleep I see entire scenes.
21.	When I think about something, images spontaneously appear.
22.	I have experienced unpleasant thoughts and ideas which I was unable to get rid of.
23.	When I observe clouds, I spontaneously see images and figures.
24.	It can happen that my imagination gets the better of me.
25.	When I close my eyes I immediately see precise patterns.
26.	It has occurred that by reading an exciting story I have experienced the scenario so vividly that it almost feels like watching a movie.
27.	It can occur that I am almost unable to distinguish between my imagined images and reality.
28.	When falling asleep I see patterns.
29.	It can occur that by smelling something, a related image spontaneously appears.
30.	In quiet hours I have imagined entire worlds which seemed to exist as reality for months.
