# Supplementary material for: Prediction of Psilocybin Response in Healthy Volunteers
Source: PLoS One. 2012 Feb 17;7(2):e30800. doi: 10.1371/journal.pone.0030800 (PMC3281871; doi:10.1371/journal.pone.0030800)
Supplement: Table S2 — Overlapping items of the EWL-K and EWL-60-S questionnaires. (PDF) [file pone.0030800.s003.pdf]

Supplementary Table S2. Overlapping items of the EWL-K and EWL-60-S questionnaires.

German	English
**Leistungsbezogene Aktivität**	**Performance-Related Activity**
tatkräftig	go-getting
eifrig	avid
aktiv	active
energisch	energetic
**Allgemeine Desaktivität**	**General Inactivation**
dösig	dozy
schläfrig	sleepy
abgespannt	weary
benebelt	blearily
energielos	shiftless
lasch	lax
kraftlos	feeble
zermürbt	harassed
müde	tired
schlaftrunken	drowsy
lahm	lame
**Extraversion/Intraversion**	**Extroversion/Introversion**
gesprächig	talkative
einsilbig*	monosyllabic*
wortkarg*	reticent*
gesellig	sociable
abgesondert*	separated*
menschenscheu*	unsociable*
zutraulich	confiding
kontaktfreudig	outgoing
**Allgemeines Wohlbehagen**	**General Well-Being**
froh	glad
unbekümmert	carefree
heiter	cheerful
lustig	amused
selbstsicher	confident
freudig	joyful
**Emotionale Gereiztheit**	**Emotional Excitability**
ärgerlich	annoyed
aufgeregt	excited
erregbar	excitable
wütend	angry
nervös	nervous
zappelig	fidgety
verletzbar	violable
erregt	agitated
Verwundbar	vulnerable
gereizt	irritable
verärgert	upset

**Angst/Deprimiertheit**	**Anxiety/Depressiveness**
betrübt	saddened
traurig	sad
beklommen	apprehensive
sorgenvoll	sorrowful
angsterfüllt	fearful
elend	miserable
furchtsam	timid

*Items scores are subtracted
