# Supplementary material for: Prediction of Psilocybin Response in Healthy Volunteers
Source: PLoS One. 2012 Feb 17;7(2):e30800. doi: 10.1371/journal.pone.0030800 (PMC3281871; doi:10.1371/journal.pone.0030800)
Supplement: Table S3 — Selection frequencies of variables at Step 1 and models at Step 2 as a result of the bootstrap model selection process (top) and pooled parameter estimates of the most frequently selected models (bottom). (PDF) [file pone.0030800.s004.pdf]

Supplementary Table S3. Selection frequencies of variables at Step 1 and models at Step 2 as a result of the bootstrap model selection process (top) and pooled parameter estimates of the most frequently selected models (bottom).

Model selection for the dependent variable $\sqrt{G\text{-}ASC}$

	Predictors	Step 1 (%)	Models selected in step 2 (the first 10 models are shown)*									
			1 (6)	2 (5)	3 (5)	4 (4)	5 (5)	6 (5)	7 (4)	8 (4)	9 (4)	10 (5)
1.	Drug dose	100.0	✓	✓	✓	✓	✓	✓	✓	✓	✓	✓
2.	Absorption (TAS)	84.8	✓	✓	✓	✓	✓	✓	✓	✓	✓	–
3.	Performance-Related Activity (EWL)	78.4	✓	✓	✓	✓	✓	–	✓	–	–	✓
4.	General Inactivation (EWL)	66.8	✓	✓	✓	✓	–	✓	–	–	✓	✓
5.	Hallucinogen-naïve	66.3	✓	–	✓	–	✓	✓	–	✓	✓	✓
6.	Emotional Excitability (EWL)	64.0	✓	✓	–	–	✓	✓	✓	✓	–	✓
7.	General Well-Being (EWL)	46.0										
8.	Anxiety-Depressiveness (EWL)	45.6										
9.	Global Severity Index (SCL-90-R)	44.2										
10.	Activity (ZKPQ)	41.4										
11.	Extroversion-Introversion (EWL)	40.6										
12.	Positron emission tomography	37.8										
13.	Alcohol frequency	36.4										
14.	Sociability (ZKPQ)	34.8										
15.	Impulsive Sensation Seeking (ZKPQ)	33.5										
16.	Aggression-Hostility (ZKPQ)	32.9										
17.	Neuroticism-Anxiety (ZKPQ)	26.5										
18.	Daily smoker	18.8										
19.	Years of education	16.4										
20.	Age	13.0										
21.	Body mass index	11.7										
22.	Time of measuring	11.2										
23.	THC frequency	9.3										
24.	Female	7.5										
Model selection frequency (%)			24.2	14.4	11.3	7.6	7.4	5.2	5.0	4.1	3.0	2.5

✓, variables included in the model; –, variables not selected.
* The number of variables in the model is given within parentheses.

Pooled parameter estimates of the best model for the dependent variable $\sqrt{G\text{-}ASC}$

Predictors	Coefficient	Standard error	*P*-value		HPD CI low	HPD CI high	*n* missings	FMI	RIV
Drug dose	0.78	0.09	0.00	***	0.60	0.96	0	0.07	0.08
Absorption (TAS)	0.35	0.12	0.00	**	0.11	0.58	312	0.44	0.76
Performance-Related Activity (EWL)	0.33	0.12	0.01	**	0.10	0.56	224	0.41	0.67
General Inactivation (EWL)	0.18	0.11	0.11		-0.04	0.40	224	0.33	0.47
Hallucinogen-naïve	0.16	0.10	0.11		-0.04	0.36	40	0.18	0.21
Emotional Excitability (EWL)	0.26	0.12	0.03	*	0.03	0.48	224	0.46	0.82

Note. HPD = Highest Posterior Density. CI = Confidence Interval. FMI = Fraction of missing information. RIV = Relative increase in variance due to missingness.

Model selection for the dependent variable $\sqrt{Oceanic\ Boundlessness}$

	Predictors	Step 1 (%)	Models selected in step 2 (the first 10 models are shown)*									
			1 (6)	2 (5)	3 (5)	4 (4)	5 (5)	6 (5)	7 (4)	8 (4)	9 (3)	10 (4)
1.	Drug dose	100.0	✓	✓	✓	✓	✓	✓	✓	✓	✓	✓
2.	Absorption (TAS)	88.7	✓	✓	✓	✓	✓	✓	✓	✓	✓	✓
3.	General Inactivation (EWL)	77.4	✓	✓	✓	✓	–	✓	–	✓	✓	–
4.	Performance-Related Activity (EWL)	76.0	✓	✓	✓	✓	✓	–	✓	–	–	–
5.	Global Severity Index (SCL-90-R)	62.4	✓	✓	–	–	✓	✓	–	–	–	✓
6.	Emotional Excitability (EWL)	62.3	✓	–	✓	–	✓	✓	✓	✓	–	✓
7.	Extroversion-Introversion (EWL)	46.5										
8.	General Well-Being (EWL)	45.4										
9.	Activity (ZKPQ)	44.9										
10.	Anxiety-Depressiveness (EWL)	42.2										
11.	Hallucinogen-naïve	42.2										
12.	Alcohol frequency	35.2										
13.	Positron emission tomography	34.9										
14.	Aggression-Hostility (ZKPQ)	34.2										
15.	Impulsive Sensation Seeking (ZKPQ)	32.8										
16.	Sociability (ZKPQ)	32.6										
17.	Neuroticism-Anxiety (ZKPQ)	27.8										
18.	Daily smoker	26.1										
19.	Age	21.1										
20.	Years of education	16.3										
21.	Body mass index	14.6										
22.	Time of measuring	12.7										
23.	THC frequency	10.4										
24.	Female	6.4										
	Model selection frequency (%)		32.1	16.6	11.8	8.8	7.0	3.3	3.2	3.0	2.4	2.0

✓, variables included in the model; –, variables not selected.
* The number of variables in the model is given within parentheses.

Pooled parameter estimates of the best model for the dependent variable $\sqrt{Oceanic\ Boundlessness}$

Predictors	Coefficient	Standard error	*P*-value		HPD CI low	HPD CI high	*n* missings	FMI	RIV
Drug dose	0.70	0.09	0.00	***	0.51	0.88	0	0.11	0.13
Absorption (TAS)	0.41	0.12	0.00	***	0.18	0.64	312	0.39	0.61
General Inactivation (EWL)	0.22	0.12	0.06		-0.01	0.46	224	0.39	0.61
Performance-Related Activity (EWL)	0.31	0.12	0.01	**	0.07	0.57	224	0.42	0.68
Global Severity Index (SCL-90-R)	-0.22	0.11	0.04	*	-0.42	-0.01	111	0.26	0.35
Emotional Excitability (EWL)	0.24	0.12	0.05	*	0.01	0.46	224	0.45	0.78

Note. HPD = Highest Posterior Density. CI = Confidence Interval. FMI = Fraction of missing information. RIV = Relative increase in variance due to missingness.

Model selection for the dependent variable log(Dread of Ego Dissolution)

	Predictors	Step 1 (%)	Models selected in step 2 (the first 10 models are shown)*									
			1 (5)	2 (6)	3 (4)	4 (5)	5 (5)	6 (4)	7 (4)	8 (3)	9 (5)	10 (3)
1.	Drug dose	100.0	✓	✓	✓	✓	✓	✓	✓	✓	✓	✓
2.	Positron emission tomography	87.0	✓	✓	✓	✓	✓	✓	✓	✓	✓	✓
3.	Time of measuring	72.4	✓	✓	✓	✓	✓	✓	✓	✓	–	–
4.	Emotional Excitability (EWL)	64.2	✓	✓	✓	✓	–	–	–	–	✓	✓
5.	Hallucinogen-naïve	60.0	✓	✓	–	–	✓	✓	–	–	✓	–
6.	General Well-Being (EWL)	50.3	–	✓	–	✓	✓	–	✓	–	✓	–
7.	Sociability (ZKPQ)	48.8										
8.	Absorption (TAS)	48.0										
9.	General Inactivation (EWL)	47.9										
10.	Performance-Related Activity (EWL)	47.1										
11.	Age	46.6										
12.	Extroversion-Introversion (EWL)	44.3										
13.	Global Severity Index (SCL-90-R)	41.9										
14.	Anxiety-Depressiveness (EWL)	39.0										
15.	Impulsive Sensation Seeking (ZKPQ)	35.2										
16.	Activity (ZKPQ)	31.8										
17.	Neuroticism-Anxiety (ZKPQ)	29.5										
18.	Aggression-Hostility (ZKPQ)	28.0										
19.	Alcohol frequency	23.8										
20.	Daily smoker	20.9										
21.	Female	19.9										
22.	Body mass index	18.8										
23.	Years of education	15.9										
24.	THC frequency	12.8										
Model selection frequency (%)			18.1	15.8	12.3	9.0	8.2	5.0	4.4	3.8	2.8	2.7

✓, variables included in the model; –, variables not selected.
* The number of variables in the model is given within parentheses.

Pooled parameter estimates of the best model for the dependent variable log(Dread of Ego Dissolution)

Predictors	Coefficient	Standard error	*P*-value		HPD CI low	HPD CI high	*n* missings	FMI	RIV
Drug dose	0.53	0.10	0.00	***	0.34	0.72	0	0.01	0.01
Positron emission tomography	0.31	0.15	0.04	*	0.02	0.59	0	0.01	0.01
Time of measuring	-0.23	0.10	0.02	*	-0.42	-0.04	0	0.01	0.01
Emotional Excitability (EWL)	0.30	0.11	0.01	**	0.08	0.51	224	0.32	0.46
Hallucinogen-naïve	0.18	0.10	0.09		-0.03	0.38	40	0.12	0.14

Note. HPD = Highest Posterior Density. CI = Confidence Interval. FMI = Fraction of missing information. RIV = Relative increase in variance due to missingness.

Model selection for the dependent variable $\sqrt{Visionary\ Restructuralization}$

	Predictors	Step 1 (%)	Models selected in step 2 (the first 10 models are shown)*									
			1 (6)	2 (7)	3 (5)	4 (5)	5 (6)	6 (6)	7 (5)	8 (4)	9 (6)	10 (4)
1.	Drug dose	100.0	✓	✓	✓	✓	✓	✓	✓	✓	✓	✓
2.	Hallucinogen-naïve	92.8	✓	✓	✓	✓	✓	✓	–	✓	✓	✓
3.	Absorption (TAS)	92.3	✓	✓	✓	✓	✓	✓	✓	✓	✓	✓
4.	Performance-Related Activity (EWL)	92.0	✓	✓	✓	✓	✓	✓	✓	✓	–	–
5.	General Inactivation (EWL)	67.3	✓	✓	✓	–	✓	–	✓	–	✓	–
6.	Emotional Excitability (EWL)	56.8	✓	✓	–	✓	–	✓	✓	–	✓	✓
7.	Alcohol frequency	54.2	–	✓	–	–	✓	✓	–	–	✓	–
8.	Extroversion-Introversion (EWL)	48.9										
9.	Sociability (ZKPQ)	47.9										
10.	Activity (ZKPQ)	46.9										
11.	Global Severity Index (SCL-90-R)	46.8										
12.	General Well-Being (EWL)	46.4										
13.	Anxiety-Depressiveness (EWL)	40.1										
14.	Positron emission tomography	38.6										
15.	Impulsive Sensation Seeking (ZKPQ)	34.8										
16.	Aggression-Hostility (ZKPQ)	30.2										
17.	Neuroticism-Anxiety (ZKPQ)	26.2										
18.	THC frequency	21.1										
19.	Years of education	16.0										
20.	Time of measuring	15.5										
21.	Age	14.0										
22.	Daily smoker	13.4										
23.	Body mass index	10.3										
24.	Female	5.4										
Model selection frequency (%)			21.7	17.9	10.1	9.2	8.6	3.1	2.8	2.4	2.1	1.8

✓, variables included in the model; –, variables not selected.
* The number of variables in the model is given within parentheses.

Pooled parameter estimates of the best model for the dependent variable $\sqrt{Visionary\ Restructuralization}$

Predictors	Coefficient	Standard error	*P*-value		HPD CI low	HPD CI high	*n* missings	FMI	RIV
Drug dose	0.71	0.09	0.00	***	0.52	0.88	0	0.08	0.09
Hallucinogen-naïve	0.22	0.11	0.04	*	0.01	0.43	40	0.25	0.32
Absorption (TAS)	0.38	0.13	0.00	**	0.12	0.63	312	0.53	1.08
Performance-Related Activity (EWL)	0.38	0.12	0.00	***	0.15	0.61	224	0.40	0.63
General Inactivation (EWL)	0.14	0.11	0.19		-0.07	0.36	224	0.31	0.44
Emotional Excitability (EWL)	0.23	0.12	0.05	*	0.00	0.46	224	0.45	0.78

Note. HPD = Highest Posterior Density. CI = Confidence Interval. FMI = Fraction of missing information. RIV = Relative increase in variance due to missingness.

Model selection for the dependent variable $\sqrt{Experience\ of\ Unity}$

	Predictors	Step 1 (%)	Models selected in step 2 (the first 10 models are shown)*									
			1 (11)	2 (8)	3 (11)	4 (11)	5 (9)	6 (10)	7 (10)	8 (12)	9 (10)	10 (11)
1.	Drug dose	100.0	✓	✓	✓	✓	✓	✓	✓	✓	✓	✓
2.	Positron emission tomography	92.2	✓	✓	✓	✓	✓	✓	✓	✓	✓	✓
3.	Absorption (TAS)	90.2	✓	✓	✓	✓	✓	✓	✓	✓	✓	✓
4.	Performance-Related Activity (EWL)	87.2	✓	–	✓	✓	✓	✓	✓	✓	✓	✓
5.	Emotional Excitability (EWL)	81.5	✓	✓	✓	✓	✓	✓	✓	✓	✓	✓
6.	Time of measuring	80.8	✓	✓	✓	✓	✓	✓	✓	✓	✓	✓
7.	General Inactivation (EWL)	79.2	✓	✓	✓	✓	–	✓	✓	✓	–	✓
8.	Global Severity Index (SCL-90-R)	76.1	✓	–	✓	✓	✓	✓	–	✓	✓	✓
9.	General Well-Being (EWL)	75.8	✓	✓	✓	✓	–	✓	✓	✓	✓	✓
10.	Hallucinogen-naïve	72.0	✓	✓	✓	✓	✓	✓	✓	✓	✓	✓
11.	Extroversion-Introversion (EWL)	68.7	–	–	–	✓	–	–	–	–	✓	–
12.	Anxiety-Depressiveness (EWL)	66.8	–	–	✓	–	✓	–	–	–	–	–
13.	Sociability (ZKPQ)	64.9	–	–	–	–	–	–	–	–	–	–
14.	Age	64.5	–	–	–	–	–	–	–	–	–	–
15.	Activity (ZKPQ)	59.6	✓	–	–	–	–	–	–	✓	–	–
16.	Aggression-Hostility (ZKPQ)	58.9	–	–	–	–	–	–	–	–	–	✓
17.	Impulsive Sensation Seeking (ZKPQ)	57.9	–	–	–	–	–	–	–	✓	–	–
18.	Neuroticism-Anxiety (ZKPQ)	55.1	–	–	–	–	–	–	✓	–	–	–
19.	Female	49.3										
20.	Alcohol frequency	41.7										
21.	Daily smoker	37.8										
22.	Years of education	31.6										
23.	Body mass index	29.9										
24.	THC frequency	23.7										
	Model selection frequency (%)		0.5	0.3	0.3	0.3	0.2	0.2	0.2	0.2	0.2	0.2

✓, variables included in the model; –, variables not selected.
* The number of variables in the model is given within parentheses.

Pooled parameter estimates of the best model for the dependent variable $\sqrt{Experience\ of\ Unity}$

Predictors	Coefficient	Standard error	*P*-value		HPD CI low	HPD CI high	*n* missings	FMI	RIV
Drug dose	0.53	0.10	0.00	***	0.33	0.73	0	0.09	0.09
Positron emission tomography	-0.12	0.21	0.58		-0.54	0.28	0	0.47	0.83
Absorption (TAS)	0.46	0.14	0.00	***	0.18	0.73	312	0.48	0.89
Performance-Related Activity (EWL)	0.27	0.14	0.04	*	0.00	0.54	224	0.34	0.50
Emotional Excitability (EWL)	0.21	0.13	0.11		-0.05	0.45	224	0.44	0.74
Time of measuring	0.07	0.10	0.51		-0.13	0.27	0	0.11	0.12
General Inactivation (EWL)	0.16	0.12	0.19		-0.08	0.39	224	0.31	0.44
Global Severity Index (SCL-90-R)	-0.22	0.12	0.06		-0.45	0.01	111	0.31	0.44
General Well-Being (EWL)	0.06	0.14	0.64		-0.20	0.34	224	0.37	0.57
Hallucinogen-naïve	0.02	0.11	0.84		-0.19	0.23	40	0.12	0.13
Activity (ZKPQ)	-0.10	0.11	0.39		-0.32	0.12	182	0.26	0.34

Note. HPD = Highest Posterior Density. CI = Confidence Interval. FMI = Fraction of missing information. RIV = Relative increase in variance due to missingness.

Model selection for the dependent variable log(Spiritual Experience)

	Predictors	Step 1 (%)	Models selected in step 2 (the first 10 models are shown)*									
			1 (11)	2 (12)	3 (10)	4 (12)	5 (13)	6 (10)	7 (12)	8 (12)	9 (9)	10 (13)
1.	Drug dose	100.0	✓	✓	✓	✓	✓	✓	✓	✓	✓	✓
2.	Absorption (TAS)	98.4	✓	✓	✓	✓	✓	✓	✓	✓	✓	✓
3.	General Inactivation (EWL)	90.7	✓	✓	✓	✓	✓	✓	✓	✓	✓	✓
4.	Emotional Excitability (EWL)	90.6	✓	✓	✓	✓	✓	✓	✓	✓	✓	✓
5.	Performance-Related Activity (EWL)	90.2	✓	✓	✓	✓	✓	✓	✓	✓	✓	✓
6.	Hallucinogen-naïve	86.6	✓	✓	✓	✓	✓	✓	✓	✓	✓	✓
7.	Time of measuring	76.9	✓	✓	✓	✓	✓	✓	✓	✓	✓	✓
8.	Activity (ZKPQ)	75.0	✓	✓	✓	✓	✓	–	✓	–	✓	✓
9.	Extroversion-Introversion (EWL)	74.4	✓	✓	–	✓	✓	–	✓	–	–	✓
10.	Global Severity Index (SCL-90-R)	73.5	✓	✓	✓	✓	✓	✓	✓	✓	✓	✓
11.	General Well-Being (EWL)	68.9	–	–	–	✓	✓	–	–	✓	–	✓
12.	Sociability (ZKPQ)	68.2	✓	✓	✓	✓	✓	✓	✓	✓	–	✓
13.	Anxiety-Depressiveness (EWL)	66.4	–	–	–	–	–	–	✓	✓	–	✓
14.	Positron emission tomography	63.6	–	–	–	–	–	✓	–	✓	–	–
15.	Aggression-Hostility (ZKPQ)	56.4	–	✓	–	–	✓	–	–	–	–	–
16.	Impulsive Sensation Seeking (ZKPQ)	53.8	–	–	–	–	–	–	–	–	–	–
17.	THC frequency	48.5										
18.	Alcohol frequency	47.4										
19.	Neuroticism-Anxiety (ZKPQ)	44.5										
20.	Female	44.2										
21.	Daily smoker	42.3										
22.	Years of education	37.9										
23.	Age	37.5										
24.	Body mass index	33.8										
	Model selection frequency (%)		0.9	0.6	0.6	0.5	0.5	0.5	0.5	0.5	0.4	0.4

✓, variables included in the model; –, variables not selected.
* The number of variables in the model is given within parentheses.

Pooled parameter estimates of the best model for the dependent variable log(Spiritual Experience)

Predictors	Coefficient	Standard error	*P*-value		HPD CI low	HPD CI high	*n* missings	FMI	RIV
Drug dose	0.41	0.10	0.00	***	0.21	0.60	0	0.16	0.19
Absorption (TAS)	0.48	0.16	0.00	**	0.17	0.78	312	0.66	1.83
General Inactivation (EWL)	0.20	0.12	0.12		-0.05	0.44	224	0.37	0.56
Emotional Excitability (EWL)	0.46	0.12	0.00	***	0.23	0.69	224	0.39	0.61
Performance-Related Activity (EWL)	0.20	0.15	0.19		-0.10	0.49	224	0.49	0.92
Hallucinogen-naïve	-0.08	0.11	0.43		-0.30	0.12	40	0.18	0.22
Time of measuring	0.27	0.10	0.01	**	0.08	0.47	0	0.18	0.21
Activity (ZKPQ)	-0.12	0.11	0.28		-0.34	0.10	182	0.30	0.42
Extroversion-Introversion (EWL)	0.02	0.14	0.88		-0.25	0.30	224	0.35	0.51
Global Severity Index (SCL-90-R)	-0.17	0.11	0.14		-0.39	0.06	111	0.34	0.50
Sociability (ZKPQ)	-0.25	0.10	0.02	*	-0.45	-0.04	182	0.24	0.31

Note. HPD = Highest Posterior Density. CI = Confidence Interval. FMI = Fraction of missing information. RIV = Relative increase in variance due to missingness.

Model selection for the dependent variable $\sqrt{Blissful\ State}$

	Predictors	Step 1 (%)	Models selected in step 2 (the first 10 models are shown)*									
			1 (13)	2 (10)	3 (12)	4 (13)	5 (11)	6 (10)	7 (13)	8 (14)	9 (14)	10 (15)
1.	Drug dose	100.0	✓	✓	✓	✓	✓	✓	✓	✓	✓	✓
2.	Performance-Related Activity (EWL)	98.2	✓	✓	✓	✓	✓	✓	✓	✓	✓	✓
3.	Hallucinogen-naïve	97.8	✓	✓	✓	✓	✓	✓	✓	✓	✓	✓
4.	Absorption (TAS)	95.8	✓	✓	✓	✓	✓	✓	✓	✓	✓	✓
5.	General Inactivation (EWL)	95.7	✓	✓	✓	✓	✓	✓	✓	✓	✓	✓
6.	Global Severity Index (SCL-90-R)	79.5	✓	✓	✓	✓	–	–	✓	✓	✓	✓
7.	Alcohol frequency	77.6	✓	✓	✓	✓	✓	✓	✓	✓	✓	✓
8.	THC frequency	76.5	✓	✓	✓	✓	✓	✓	✓	✓	✓	✓
9.	Emotional Excitability (EWL)	75.5	✓	✓	✓	✓	✓	✓	✓	✓	✓	✓
10.	General Well-Being (EWL)	72.9	✓	–	✓	✓	✓	–	✓	✓	✓	✓
11.	Extroversion-Introversion (EWL)	71.5	✓	–	–	✓	–	–	–	✓	✓	–
12.	Sociability (ZKPQ)	67.7	–	–	–	–	–	–	–	–	–	✓
13.	Positron emission tomography	64.9	–	✓	–	✓	–	–	–	–	✓	✓
14.	Anxiety-Depressiveness (EWL)	64.5	–	–	–	–	–	–	✓	✓	–	✓
15.	Activity (ZKPQ)	62.3	–	–	–	–	–	–	–	–	–	–
16.	Impulsive Sensation Seeking (ZKPQ)	57.9	–	–	–	✓	–	–	–	–	–	–
17.	Years of education	57.8	✓	–	✓	–	✓	✓	✓	✓	✓	✓
18.	Age	56.9	✓	–	✓	–	✓	✓	✓	✓	✓	✓
19.	Aggression-Hostility (ZKPQ)	48.9										
20.	Daily smoker	48.7										
21.	Neuroticism-Anxiety (ZKPQ)	48.5										
22.	Time of measuring	27.1										
23.	Body mass index	22.1										
24.	Female	20.9										
Model selection frequency (%)			0.4	0.4	0.4	0.3	0.3	0.3	0.3	0.3	0.3	0.3

✓, variables included in the model; –, variables not selected.
* The number of variables in the model is given within parentheses.

Pooled parameter estimates of the best model for the dependent variable $\sqrt{Blissful\ State}$

Predictors	Coefficient	Standard error	*P*-value		HPD CI low	HPD CI high	*n* missings	FMI	RIV
Drug dose	0.54	0.10	0.00	***	0.34	0.74	0	0.10	0.10
Performance-Related Activity (EWL)	0.30	0.14	0.03	*	0.02	0.58	224	0.34	0.50
Hallucinogen-naïve	0.17	0.11	0.11		-0.04	0.39	40	0.11	0.12
Absorption (TAS)	0.02	0.16	0.89		-0.30	0.33	312	0.46	0.82
General Inactivation (EWL)	0.24	0.13	0.08		-0.03	0.49	224	0.38	0.59
Global Severity Index (SCL-90-R)	-0.26	0.11	0.02	*	-0.48	-0.04	111	0.22	0.27
Alcohol frequency	0.16	0.11	0.14		-0.06	0.38	83	0.24	0.31
THC sometimes vs rarely	0.33	0.11	0.00	**	0.11	0.55	47	0.09	0.10
THC rarely vs never	0.02	0.15	0.89		-0.28	0.32	47	0.23	0.28
Emotional Excitability (EWL)	0.19	0.12	0.11		-0.04	0.42	224	0.32	0.46
General Well-Being (EWL)	0.22	0.16	0.15		-0.09	0.53	224	0.44	0.74
Extroversion-Introversion (EWL)	-0.14	0.19	0.45		-0.51	0.22	224	0.53	1.07
Years of education	-0.19	0.13	0.13		-0.45	0.06	0	0.24	0.30
Age	0.22	0.11	0.06		-0.01	0.44	0	0.15	0.17

Note. HPD = Highest Posterior Density. CI = Confidence Interval. FMI = Fraction of missing information. RIV = Relative increase in variance due to missingness.

Model selection for the dependent variable log(Insightfulness)

			Models selected in step 2 (the first 10 models are shown)*									
	Predictors	Step 1 (%)	1 (8)	2 (8)	3 (9)	4 (9)	5 (9)	6 (8)	7 (8)	8 (10)	9 (8)	10 (7)
1.	Drug dose	100.0	✓	✓	✓	✓	✓	✓	✓	✓	✓	✓
2.	Absorption (TAS)	94.9	✓	✓	✓	✓	✓	✓	✓	✓	✓	✓
3.	Performance-Related Activity (EWL)	91.3	✓	✓	✓	✓	✓	✓	✓	✓	✓	✓
4.	General Inactivation (EWL)	89.7	✓	✓	✓	✓	✓	✓	✓	✓	✓	✓
5.	Hallucinogen-naïve	87.8	✓	✓	✓	✓	✓	✓	✓	✓	✓	✓
6.	Emotional Excitability (EWL)	84.2	✓	✓	✓	✓	✓	✓	✓	✓	✓	✓
7.	Activity (ZKPQ)	72.1	✓	✓	✓	–	✓	–	–	✓	–	–
8.	Positron emission tomography	71.8	✓	–	✓	✓	✓	–	–	–	✓	–
9.	Neuroticism-Anxiety (ZKPQ)	66.9	–	✓	–	✓	✓	✓	✓	✓	–	✓
10.	General Well-Being (EWL)	65.9	–	–	–	–	–	–	–	–	–	–
11.	Anxiety-Depressiveness (EWL)	64.6	–	–	–	–	–	–	✓	–	–	–
12.	Extroversion-Introversion (EWL)	62.2	–	–	–	–	–	–	–	–	–	–
13.	Impulsive Sensation Seeking (ZKPQ)	61.4	–	–	–	–	–	–	–	✓	–	–
14.	Aggression-Hostility (ZKPQ)	56.1	–	–	–	✓	–	–	–	–	–	–
15.	Female	55.7	–	–	✓	–	–	✓	–	✓	✓	–
16.	Global Severity Index (SCL-90-R)	55.2	–	–	–	–	–	–	–	–	–	–
17.	Sociability (ZKPQ)	47.7										
18.	Alcohol frequency	45.2										
19.	THC frequency	34.2										
20.	Daily smoker	30.6										
21.	Age	30.1										
22.	Years of education	29.7										
23.	Time of measuring	28.8										
24.	Body mass index	24.3										
	Model selection frequency (%)		0.6	0.5	0.5	0.5	0.4	0.4	0.4	0.4	0.4	0.4

✓, variables included in the model; –, variables not selected.
* The number of variables in the model is given within parentheses.

Pooled parameter estimates of the best model for the dependent variable log(Insightfulness)

Predictors	Coefficient	Standard error	*P*-value		HPD CI low	HPD CI high	*n* missings	FMI	RIV
Drug dose	0.48	0.10	0.00	***	0.29	0.67	0	0.07	0.08
Absorption (TAS)	0.36	0.13	0.00	**	0.11	0.60	312	0.37	0.58
Performance-Related Activity (EWL)	0.25	0.13	0.06		-0.01	0.51	224	0.43	0.72
General Inactivation (EWL)	0.21	0.12	0.09		-0.03	0.44	224	0.34	0.50
Hallucinogen-naïve	0.20	0.11	0.06		-0.01	0.42	40	0.16	0.19
Emotional Excitability (EWL)	0.26	0.11	0.02	*	0.04	0.48	224	0.32	0.46
Activity (ZKPQ)	-0.20	0.12	0.10		-0.42	0.04	182	0.36	0.53
Positron emission tomography	0.19	0.18	0.27		-0.16	0.53	0	0.31	0.43

Note. HPD = Highest Posterior Density. CI = Confidence Interval. FMI = Fraction of missing information. RIV = Relative increase in variance due to missingness.

Model selection for the dependent variable log(Disembodiment)

	Predictors	Step 1 (%)	Models selected in step 2 (the first 10 models are shown)*									
			1 (16)	2 (15)	3 (15)	4 (14)	5 (13)	6 (14)	7 (15)	8 (15)	9 (17)	10 (14)
1.	Drug dose	100.0	✓	✓	✓	✓	✓	✓	✓	✓	✓	✓
2.	Absorption (TAS)	99.5	✓	✓	✓	✓	✓	✓	✓	✓	✓	✓
3.	General Inactivation (EWL)	95.2	✓	✓	✓	✓	✓	✓	✓	✓	✓	✓
4.	Emotional Excitability (EWL)	94.8	✓	✓	✓	✓	✓	✓	✓	✓	✓	✓
5.	Performance-Related Activity (EWL)	94.3	✓	✓	✓	✓	✓	✓	✓	✓	✓	✓
6.	Hallucinogen-naïve	94.3	✓	✓	✓	✓	✓	✓	✓	✓	✓	✓
7.	Extroversion-Introversion (EWL)	86.1	✓	✓	✓	✓	✓	✓	✓	✓	✓	✓
8.	Positron emission tomography	84.1	✓	✓	✓	–	✓	✓	✓	✓	✓	✓
9.	General Well-Being (EWL)	82.0	✓	✓	✓	✓	✓	✓	✓	✓	✓	✓
10.	Time of measuring	81.2	✓	✓	✓	✓	✓	✓	✓	✓	✓	✓
11.	Global Severity Index (SCL-90-R)	81.0	✓	✓	✓	✓	✓	✓	✓	✓	✓	✓
12.	Anxiety-Depressiveness (EWL)	81.0	✓	✓	✓	✓	✓	✓	–	✓	✓	✓
13.	Activity (ZKPQ)	80.5	✓	–	✓	✓	–	✓	✓	✓	✓	–
14.	Sociability (ZKPQ)	80.5	✓	✓	✓	✓	✓	✓	✓	✓	✓	✓
15.	Aggression-Hostility (ZKPQ)	70.5	✓	✓	–	–	–	–	✓	✓	✓	–
16.	Impulsive Sensation Seeking (ZKPQ)	67.1	✓	✓	✓	✓	–	–	✓	–	✓	✓
17.	THC frequency	63.2	–	–	–	–	–	–	–	–	✓	–
18.	Alcohol frequency	60.3	–	–	–	–	–	–	–	–	–	–
19.	Neuroticism-Anxiety (ZKPQ)	58.0	–	–	–	–	–	–	–	–	–	–
20.	Female	54.5	–	–	–	–	–	–	–	–	–	–
21.	Daily smoker	52.9	–	–	–	–	–	–	–	–	–	–
22.	Years of education	47.7										
23.	Age	45.4										
24.	Body mass index	40.9										
Model selection frequency (%)			0.9	0.5	0.4	0.4	0.4	0.4	0.4	0.4	0.4	0.3

✓, variables included in the model; –, variables not selected.
* The number of variables in the model is given within parentheses.

Pooled parameter estimates of the best model for the dependent variable log(Disembodiment)

Predictors	Coefficient	Standard error	*P*-value		HPD CI low	HPD CI high	*n* missings	FMI	RIV
Drug dose	0.41	0.10	0.00	***	0.21	0.61	0	0.05	0.05
Absorption (TAS)	0.27	0.13	0.04	*	0.01	0.53	312	0.29	0.39
General Inactivation (EWL)	0.10	0.14	0.48		-0.19	0.38	224	0.42	0.69
Emotional Excitability (EWL)	0.12	0.14	0.38		-0.14	0.40	224	0.39	0.61
Performance-Related Activity (EWL)	0.24	0.15	0.10		-0.05	0.54	224	0.36	0.55
Hallucinogen-naïve	0.25	0.11	0.03	*	0.03	0.46	40	0.12	0.14
Extroversion-Introversion (EWL)	-0.11	0.20	0.58		-0.50	0.27	224	0.47	0.85
Positron emission tomography	0.24	0.19	0.21		-0.15	0.61	0	0.29	0.39
General Well-Being (EWL)	0.14	0.20	0.46		-0.26	0.52	224	0.39	0.62
Time of measuring	-0.07	0.10	0.50		-0.27	0.13	0	0.04	0.05
Global Severity Index (SCL-90-R)	-0.16	0.13	0.21		-0.40	0.09	111	0.33	0.48
Anxiety-Depressiveness (EWL)	0.08	0.14	0.58		-0.20	0.36	224	0.31	0.43
Activity (ZKPQ)	0.06	0.12	0.59		-0.17	0.29	182	0.22	0.27
Sociability (ZKPQ)	0.04	0.13	0.74		-0.22	0.30	182	0.34	0.50
Aggression-Hostility (ZKPQ)	0.16	0.13	0.21		-0.09	0.41	182	0.36	0.55
Impulsive Sensation Seeking (ZKPQ)	0.00	0.16	1.00		-0.30	0.31	182	0.32	0.45

Note. HPD = Highest Posterior Density. CI = Confidence Interval. FMI = Fraction of missing information. RIV = Relative increase in variance due to missingness.

Model selection for the dependent variable $\sqrt{Impaired\ Control\ and\ Cognition}$

	Predictors	Step 1 (%)	Models selected in step 2 (the first 10 models are shown)*									
			1 (19)	2 (18)	3 (20)	4 (17)	5 (18)	6 (19)	7 (20)	8 (18)	9 (18)	10 (17)
1.	Drug dose	100.0	✓	✓	✓	✓	✓	✓	✓	✓	✓	✓
2.	Hallucinogen-naïve	99.4	✓	✓	✓	✓	✓	✓	✓	✓	✓	✓
3.	Performance-Related Activity (EWL)	99.2	✓	✓	✓	✓	✓	✓	✓	✓	✓	✓
4.	Absorption (TAS)	98.3	✓	✓	✓	✓	✓	✓	✓	✓	✓	✓
5.	General Inactivation (EWL)	97.3	✓	✓	✓	✓	✓	✓	✓	✓	✓	✓
6.	Global Severity Index (SCL-90-R)	90.5	✓	✓	✓	✓	✓	✓	✓	✓	✓	✓
7.	Time of measuring	87.5	✓	✓	✓	✓	✓	✓	✓	–	✓	✓
8.	Extroversion-Introversion (EWL)	84.7	✓	✓	✓	✓	✓	✓	✓	✓	✓	✓
9.	General Well-Being (EWL)	84.7	✓	✓	✓	✓	✓	✓	✓	✓	✓	✓
10.	Emotional Excitability (EWL)	84.5	✓	✓	✓	✓	✓	✓	✓	✓	✓	✓
11.	Anxiety-Depressiveness (EWL)	82.6	✓	✓	✓	✓	✓	✓	✓	✓	✓	✓
12.	Alcohol frequency	82.3	✓	✓	✓	✓	✓	✓	✓	✓	✓	✓
13.	Positron emission tomography	80.8	✓	✓	✓	✓	✓	✓	✓	✓	✓	–
14.	Sociability (ZKPQ)	80.3	✓	✓	✓	✓	✓	✓	✓	✓	✓	✓
15.	THC frequency	80.1	✓	✓	✓	✓	✓	✓	✓	✓	✓	✓
16.	Age	79.8	✓	✓	✓	✓	✓	✓	✓	✓	–	✓
17.	Activity (ZKPQ)	73.5	✓	✓	✓	–	–	–	✓	✓	✓	–
18.	Impulsive Sensation Seeking (ZKPQ)	72.7	✓	✓	✓	✓	✓	✓	✓	✓	✓	✓
19.	Aggression-Hostility (ZKPQ)	66.1	–	–	–	–	–	✓	✓	–	✓	–
20.	Neuroticism-Anxiety (ZKPQ)	64.5	–	–	✓	–	–	–	–	–	–	–
21.	Years of education	63.2	✓	–	✓	–	✓	✓	✓	✓	–	✓
22.	Daily smoker	61.9	–	–	–	–	–	–	–	–	–	–
23.	Body mass index	42.2										
24.	Female	42.1										
	Model selection frequency (%)		0.4	0.3	0.3	0.3	0.2	0.2	0.2	0.2	0.2	0.2

✓, variables included in the model; –, variables not selected.
* The number of variables in the model is given within parentheses.

Pooled parameter estimates of the best model for the dependent variable $\sqrt{Impaired\ Control\ and\ Cognition}$

Predictors	Coefficient	Standard error	*P*-value		HPD CI low	HPD CI high	*n* missings	FMI	RIV
Drug dose	0.49	0.10	0.00	***	0.28	0.69	0	0.05	0.06
Hallucinogen-naïve	0.17	0.11	0.14		-0.05	0.38	40	0.08	0.09
Performance-Related Activity (EWL)	0.15	0.16	0.36		-0.18	0.46	224	0.48	0.89
Absorption (TAS)	0.06	0.22	0.73		-0.39	0.47	312	0.65	1.74
General Inactivation (EWL)	-0.01	0.15	0.94		-0.29	0.29	224	0.45	0.78
Global Severity Index (SCL-90-R)	0.13	0.13	0.31		-0.12	0.38	111	0.33	0.47
Time of measuring	-0.22	0.10	0.03	*	-0.43	-0.03	0	0.04	0.04
Extroversion-Introversion (EWL)	-0.10	0.21	0.64		-0.50	0.30	224	0.50	0.95
General Well-Being (EWL)	0.00	0.23	0.97		-0.45	0.44	224	0.55	1.18
Emotional Excitability (EWL)	0.06	0.14	0.67		-0.22	0.34	224	0.40	0.63
Anxiety-Depressiveness (EWL)	0.22	0.16	0.15		-0.08	0.53	224	0.41	0.66
Alcohol frequency	0.13	0.12	0.26		-0.10	0.36	83	0.25	0.33
Positron emission tomography	0.09	0.18	0.62		-0.27	0.46	0	0.20	0.25
Sociability (ZKPQ)	0.10	0.14	0.45		-0.16	0.37	182	0.29	0.40
THC sometimes vs rarely	0.05	0.12	0.67		-0.18	0.28	47	0.07	0.08
THC rarely vs never	-0.17	0.15	0.26		-0.48	0.13	47	0.21	0.26
Age	-0.27	0.13	0.04	*	-0.52	-0.02	0	0.20	0.24
Activity (ZKPQ)	0.06	0.13	0.66		-0.21	0.32	182	0.38	0.58
Impulsive Sensation Seeking (ZKPQ)	-0.09	0.18	0.60		-0.43	0.26	182	0.45	0.77
Years of education	-0.04	0.15	0.81		-0.34	0.26	0	0.37	0.56

Note. HPD = Highest Posterior Density. CI = Confidence Interval. FMI = Fraction of missing information. RIV = Relative increase in variance due to missingness.

Model selection for the dependent variable $\frac{-1}{\sqrt{Anxiety}}$

	Predictors	Step 1 (%)	Models selected in step 2 (the first 10 models are shown)*									
			1 (17)	2 (15)	3 (18)	4 (15)	5 (16)	6 (16)	7 (15)	8 (16)	9 (15)	10 (14)
1.	Drug dose	100.0	✓	✓	✓	✓	✓	✓	✓	✓	✓	✓
2.	Positron emission tomography	99.7	✓	✓	✓	✓	✓	✓	✓	✓	✓	✓
3.	Emotional Excitability (EWL)	98.8	✓	✓	✓	✓	✓	✓	✓	✓	✓	✓
4.	Absorption (TAS)	97.9	✓	✓	✓	✓	✓	✓	✓	✓	✓	✓
5.	Performance-Related Activity (EWL)	96.1	✓	✓	✓	✓	✓	✓	✓	✓	✓	✓
6.	General Inactivation (EWL)	93.2	✓	✓	✓	✓	✓	✓	✓	✓	✓	✓
7.	Hallucinogen-naïve	91.5	✓	✓	✓	✓	✓	✓	✓	✓	✓	✓
8.	Neuroticism-Anxiety (ZKPQ)	81.4	✓	✓	✓	–	✓	✓	✓	✓	✓	✓
9.	THC frequency	81.3	✓	✓	✓	✓	✓	✓	✓	✓	✓	✓
10.	General Well-Being (EWL)	80.7	✓	–	✓	✓	✓	–	✓	✓	✓	✓
11.	Activity (ZKPQ)	80.1	✓	✓	✓	✓	✓	✓	✓	✓	✓	✓
12.	Anxiety-Depressiveness (EWL)	77.6	✓	✓	✓	✓	✓	✓	✓	✓	–	–
13.	Impulsive Sensation Seeking (ZKPQ)	76.1	✓	–	✓	✓	✓	✓	✓	✓	✓	✓
14.	Extroversion-Introversion (EWL)	75.0	✓	✓	✓	✓	–	–	–	✓	✓	–
15.	Aggression-Hostility (ZKPQ)	73.1	✓	✓	✓	–	✓	✓	✓	✓	–	✓
16.	Global Severity Index (SCL-90-R)	69.4	–	–	✓	–	✓	–	–	–	✓	–
17.	Sociability (ZKPQ)	69.0	✓	✓	✓	✓	–	✓	✓	✓	–	–
18.	Female	58.9	✓	✓	✓	✓	✓	✓	–	–	✓	✓
19.	Time of measuring	56.8	–	–	–	–	–	✓	–	–	–	–
20.	Alcohol frequency	56.3	–	–	–	–	–	–	–	–	–	–
21.	Daily smoker	48.9										
22.	Age	48.3										
23.	Years of education	46.8										
24.	Body mass index	41.1										
	Model selection frequency (%)		0.3	0.2	0.2	0.2	0.2	0.2	0.2	0.2	0.2	0.2

✓, variables included in the model; –, variables not selected.
* The number of variables in the model is given within parentheses.

Pooled parameter estimates of the best model for the dependent variable $\frac{-1}{\sqrt{Anxiety}}$

Predictors	Coefficient	Standard error	*P*-value		HPD CI low	HPD CI high	*n* missings	FMI	RIV
Drug dose	0.30	0.10	0.00	**	0.10	0.49	0	0.05	0.05
Positron emission tomography	0.65	0.18	0.00	***	0.30	1.00	0	0.17	0.20
Emotional Excitability (EWL)	0.52	0.15	0.00	***	0.23	0.83	224	0.50	0.94
Absorption (TAS)	0.07	0.14	0.63		-0.21	0.35	312	0.44	0.75
Performance-Related Activity (EWL)	0.11	0.15	0.44		-0.18	0.39	224	0.39	0.63
General Inactivation (EWL)	0.06	0.14	0.67		-0.20	0.33	224	0.37	0.55
Hallucinogen-naïve	0.12	0.11	0.27		-0.09	0.34	40	0.14	0.17
Neuroticism-Anxiety (ZKPQ)	-0.13	0.15	0.40		-0.43	0.15	182	0.50	0.95
THC sometimes vs rarely	-0.23	0.12	0.07		-0.46	0.02	47	0.23	0.28
THC rarely vs never	0.21	0.15	0.16		-0.08	0.49	47	0.14	0.15
General Well-Being (EWL)	-0.14	0.20	0.48		-0.53	0.25	224	0.41	0.67
Activity (ZKPQ)	-0.06	0.14	0.68		-0.33	0.21	182	0.43	0.72
Anxiety-Depressiveness (EWL)	-0.02	0.14	0.88		-0.28	0.25	224	0.20	0.24
Impulsive Sensation Seeking (ZKPQ)	-0.06	0.16	0.71		-0.38	0.26	182	0.40	0.65
Extroversion-Introversion (EWL)	-0.03	0.18	0.84		-0.40	0.32	224	0.40	0.63
Aggression-Hostility (ZKPQ)	-0.10	0.13	0.44		-0.35	0.15	182	0.32	0.45
Sociability (ZKPQ)	0.17	0.12	0.17		-0.07	0.41	182	0.23	0.29
Female	0.04	0.11	0.74		-0.19	0.25	0	0.23	0.29

Note. HPD = Highest Posterior Density. CI = Confidence Interval. FMI = Fraction of missing information. RIV = Relative increase in variance due to missingness.

Model selection for the dependent variable $\sqrt{Complex\ Imagery}$

	Predictors	Step 1 (%)	Models selected in step 2 (the first 10 models are shown)*									
			1 (18)	2 (17)	3 (16)	4 (17)	5 (16)	6 (17)	7 (16)	8 (16)	9 (17)	10 (15)
1.	Drug dose	100.0	✓	✓	✓	✓	✓	✓	✓	✓	✓	✓
2.	Absorption (TAS)	98.6	✓	✓	✓	✓	✓	✓	✓	✓	✓	✓
3.	Performance-Related Activity (EWL)	97.2	✓	✓	✓	✓	✓	✓	✓	✓	✓	✓
4.	Positron emission tomography	96.4	✓	✓	✓	✓	✓	✓	✓	✓	✓	✓
5.	Hallucinogen-naïve	95.3	✓	✓	✓	✓	✓	✓	✓	✓	✓	✓
6.	General Inactivation (EWL)	91.3	✓	✓	✓	✓	✓	✓	✓	✓	✓	✓
7.	Emotional Excitability (EWL)	91.3	✓	✓	✓	✓	✓	✓	✓	✓	✓	✓
8.	Global Severity Index (SCL-90-R)	90.0	✓	✓	✓	✓	✓	✓	✓	✓	✓	✓
9.	Extroversion-Introversion (EWL)	87.5	✓	✓	✓	✓	✓	✓	✓	✓	✓	✓
10.	General Well-Being (EWL)	85.9	✓	✓	✓	✓	✓	✓	✓	✓	✓	✓
11.	Time of measuring	84.7	✓	✓	✓	✓	✓	✓	✓	✓	✓	✓
12.	Sociability (ZKPQ)	79.2	✓	✓	✓	✓	✓	✓	✓	✓	✓	✓
13.	Anxiety-Depressiveness (EWL)	78.1	✓	✓	✓	✓	✓	✓	✓	✓	✓	✓
14.	Alcohol frequency	77.1	✓	✓	✓	–	✓	✓	✓	–	✓	✓
15.	Impulsive Sensation Seeking (ZKPQ)	75.1	✓	✓	✓	✓	✓	✓	–	✓	✓	–
16.	Activity (ZKPQ)	73.2	✓	–	✓	✓	–	✓	✓	✓	✓	✓
17.	Aggression-Hostility (ZKPQ)	70.0	✓	✓	–	✓	✓	✓	–	✓	–	–
18.	Age	69.2	–	–	–	–	–	–	–	–	–	–
19.	Neuroticism-Anxiety (ZKPQ)	64.6	✓	✓	–	✓	–	–	✓	–	✓	–
20.	Female	52.0	–	–	–	–	–	–	–	–	–	–
21.	Daily smoker	48.7										
22.	THC frequency	45.0										
23.	Years of education	41.3										
24.	Body mass index	39.8										
Model selection frequency (%)			0.6	0.6	0.5	0.5	0.4	0.4	0.4	0.4	0.4	0.4

✓, variables included in the model; –, variables not selected.
* The number of variables in the model is given within parentheses.

Pooled parameter estimates of the best model for the dependent variable $\sqrt{Complex\ Imagery}$

Predictors	Coefficient	Standard error	*P*-value		HPD CI low	HPD CI high	*n* missings	FMI	RIV
Drug dose	0.47	0.10	0.00	***	0.26	0.67	0	0.12	0.14
Absorption (TAS)	0.45	0.15	0.00	**	0.15	0.73	312	0.49	0.91
Performance-Related Activity (EWL)	0.40	0.15	0.00	**	0.12	0.69	224	0.38	0.58
Positron emission tomography	0.03	0.22	0.85		-0.43	0.45	0	0.51	0.99
Hallucinogen-naïve	0.20	0.12	0.08		-0.02	0.43	40	0.24	0.31
General Inactivation (EWL)	0.08	0.14	0.56		-0.18	0.34	224	0.37	0.57
Emotional Excitability (EWL)	0.22	0.14	0.12		-0.06	0.47	224	0.39	0.62
Global Severity Index (SCL-90-R)	-0.25	0.12	0.04	*	-0.49	-0.01	111	0.22	0.27
Extroversion-Introversion (EWL)	-0.26	0.18	0.12		-0.62	0.07	224	0.35	0.51
General Well-Being (EWL)	0.01	0.22	0.94		-0.41	0.43	224	0.52	1.04
Time of measuring	0.14	0.10	0.16		-0.06	0.34	0	0.08	0.09
Sociability (ZKPQ)	0.13	0.13	0.31		-0.12	0.39	182	0.38	0.58
Anxiety-Depressiveness (EWL)	0.07	0.15	0.65		-0.22	0.35	224	0.36	0.55
Alcohol frequency	0.28	0.11	0.01	*	0.06	0.50	83	0.23	0.29
Impulsive Sensation Seeking (ZKPQ)	-0.05	0.17	0.75		-0.38	0.28	182	0.47	0.85
Activity (ZKPQ)	-0.03	0.12	0.79		-0.27	0.21	182	0.34	0.49
Aggression-Hostility (ZKPQ)	0.06	0.12	0.62		-0.18	0.29	182	0.24	0.31
Neuroticism-Anxiety (ZKPQ)	-0.03	0.13	0.81		-0.29	0.22	182	0.22	0.28

Note. HPD = Highest Posterior Density. CI = Confidence Interval. FMI = Fraction of missing information. RIV = Relative increase in variance due to missingness.

Model selection for the dependent variable $\sqrt{Elementary\ Imagery}$

	Predictors	Step 1 (%)	Models selected in step 2 (the first 10 models are shown)*									
			1 (17)	2 (17)	3 (16)	4 (18)	5 (18)	6 (18)	7 (19)	8 (18)	9 (16)	10 (17)
1.	Drug dose	100.0	✓	✓	✓	✓	✓	✓	✓	✓	✓	✓
2.	Absorption (TAS)	99.9	✓	✓	✓	✓	✓	✓	✓	✓	✓	✓
3.	Performance-Related Activity (EWL)	98.4	✓	✓	✓	✓	✓	✓	✓	✓	✓	✓
4.	Hallucinogen-naïve	97.9	✓	✓	✓	✓	✓	✓	✓	✓	✓	✓
5.	General Inactivation (EWL)	97.6	✓	✓	✓	✓	✓	✓	✓	✓	✓	✓
6.	Emotional Excitability (EWL)	96.6	✓	✓	✓	✓	✓	✓	✓	✓	✓	✓
7.	Extroversion-Introversion (EWL)	93.6	✓	✓	✓	✓	✓	✓	✓	✓	✓	✓
8.	Sociability (ZKPQ)	91.7	✓	✓	✓	✓	✓	✓	✓	✓	✓	✓
9.	Positron emission tomography	90.5	✓	✓	✓	✓	✓	✓	✓	✓	✓	✓
10.	General Well-Being (EWL)	90.5	✓	✓	✓	✓	✓	✓	✓	✓	✓	✓
11.	Activity (ZKPQ)	89.8	✓	✓	✓	✓	✓	✓	✓	✓	✓	✓
12.	Time of measuring	89.6	✓	✓	✓	✓	✓	✓	✓	✓	✓	✓
13.	Anxiety-Depressiveness (EWL)	89.3	✓	✓	✓	✓	✓	✓	✓	✓	✓	✓
14.	Global Severity Index (SCL-90-R)	85.1	✓	✓	✓	✓	✓	✓	✓	✓	✓	✓
15.	Aggression-Hostility (ZKPQ)	78.8	✓	✓	✓	✓	✓	✓	✓	✓	✓	✓
16.	Impulsive Sensation Seeking (ZKPQ)	77.5	✓	✓	✓	✓	✓	✓	✓	✓	–	✓
17.	Alcohol frequency	71.0	–	✓	–	✓	✓	–	✓	–	–	–
18.	Neuroticism-Anxiety (ZKPQ)	70.6	✓	–	–	–	✓	–	–	✓	✓	–
19.	THC frequency	69.1	–	–	–	–	–	✓	✓	✓	–	–
20.	Daily smoker	65.5	–	–	–	✓	–	✓	✓	–	–	✓
21.	Female	58.4	–	–	–	–	–	–	–	–	–	–
22.	Years of education	54.0	–	–	–	–	–	–	–	–	–	–
23.	Age	53.3	–	–	–	–	–	–	–	–	–	–
24.	Body mass index	48.6										
	Model selection frequency (%)		1.1	0.8	0.6	0.6	0.6	0.5	0.5	0.5	0.4	0.4

✓, variables included in the model; –, variables not selected.
* The number of variables in the model is given within parentheses.

Pooled parameter estimates of the best model for the dependent variable $\sqrt{Elementary\ Imagery}$

Predictors	Coefficient	Standard error	*P*-value		HPD CI low	HPD CI high	*n* missings	FMI	RIV
Drug dose	0.56	0.11	0.00	***	0.34	0.77	0	0.15	0.17
Absorption (TAS)	0.36	0.14	0.01	**	0.09	0.64	312	0.40	0.63
Performance-Related Activity (EWL)	0.45	0.14	0.00	***	0.18	0.73	224	0.29	0.39
Hallucinogen-naïve	0.13	0.12	0.24		-0.09	0.36	40	0.23	0.29
General Inactivation (EWL)	0.05	0.13	0.74		-0.20	0.31	224	0.30	0.42
Emotional Excitability (EWL)	0.18	0.14	0.23		-0.12	0.45	224	0.45	0.78
Extroversion-Introversion (EWL)	-0.15	0.19	0.44		-0.54	0.21	224	0.46	0.81
Sociability (ZKPQ)	0.21	0.13	0.11		-0.05	0.46	182	0.36	0.54
Positron emission tomography	-0.15	0.21	0.50		-0.58	0.26	0	0.46	0.81
General Well-Being (EWL)	-0.04	0.21	0.84		-0.44	0.35	224	0.46	0.81
Activity (ZKPQ)	-0.14	0.12	0.26		-0.38	0.10	182	0.29	0.39
Time of measuring	0.22	0.10	0.03	*	0.02	0.42	0	0.06	0.07
Anxiety-Depressiveness (EWL)	0.02	0.14	0.89		-0.27	0.29	224	0.28	0.38
Global Severity Index (SCL-90-R)	-0.09	0.13	0.49		-0.35	0.17	111	0.28	0.37
Aggression-Hostility (ZKPQ)	-0.10	0.12	0.42		-0.33	0.14	182	0.21	0.26
Impulsive Sensation Seeking (ZKPQ)	-0.09	0.17	0.62		-0.42	0.24	182	0.44	0.74
Neuroticism-Anxiety (ZKPQ)	-0.15	0.14	0.30		-0.43	0.13	182	0.32	0.45

Note. HPD = Highest Posterior Density. CI = Confidence Interval. FMI = Fraction of missing information. RIV = Relative increase in variance due to missingness.

Model selection for the dependent variable log(Audio-Visual Synesthesiae)

	Predictors	Step 1 (%)	Models selected in step 2 (the first 10 models are shown)*									
			1 (20)	2 (19)	3 (21)	4 (19)	5 (19)	6 (19)	7 (18)	8 (19)	9 (23)	10 (18)
1.	Drug dose	100.0	✓	✓	✓	✓	✓	✓	✓	✓	✓	✓
2.	Positron emission tomography	99.9	✓	✓	✓	✓	✓	✓	✓	✓	✓	✓
3.	Emotional Excitability (EWL)	99.6	✓	✓	✓	✓	✓	✓	✓	✓	✓	✓
4.	Absorption (TAS)	99.2	✓	✓	✓	✓	✓	✓	✓	✓	✓	✓
5.	Performance-Related Activity (EWL)	97.7	✓	✓	✓	✓	✓	✓	✓	✓	✓	✓
6.	Hallucinogen-naïve	96.6	✓	✓	✓	✓	✓	✓	✓	✓	✓	✓
7.	General Inactivation (EWL)	96.0	✓	✓	✓	✓	✓	✓	✓	✓	✓	✓
8.	Alcohol frequency	91.4	✓	✓	✓	✓	✓	✓	✓	✓	✓	✓
9.	General Well-Being (EWL)	91.2	✓	✓	✓	✓	✓	✓	✓	✓	✓	✓
10.	Extroversion-Introversion (EWL)	88.8	✓	✓	✓	✓	✓	✓	✓	✓	✓	✓
11.	Activity (ZKPQ)	88.0	✓	✓	✓	✓	✓	✓	✓	✓	✓	✓
12.	Neuroticism-Anxiety (ZKPQ)	87.1	✓	✓	✓	✓	✓	✓	✓	✓	✓	✓
13.	Anxiety-Depressiveness (EWL)	87.1	✓	✓	✓	✓	✓	✓	✓	–	✓	✓
14.	Impulsive Sensation Seeking (ZKPQ)	84.8	✓	✓	✓	✓	✓	✓	✓	✓	✓	✓
15.	THC frequency	84.5	✓	✓	✓	✓	✓	✓	–	✓	✓	✓
16.	Sociability (ZKPQ)	83.4	✓	✓	✓	✓	✓	✓	✓	✓	✓	✓
17.	Aggression-Hostility (ZKPQ)	81.3	✓	✓	✓	✓	✓	–	✓	✓	✓	✓
18.	Time of measuring	79.3	✓	✓	✓	–	✓	✓	✓	✓	✓	–
19.	Global Severity Index (SCL-90-R)	77.8	✓	–	✓	✓	✓	✓	✓	✓	✓	–
20.	Years of education	68.8	–	–	✓	–	–	–	–	–	✓	–
21.	Age	65.0	–	–	–	–	–	–	–	–	✓	–
22.	Daily smoker	62.6	–	–	–	–	–	–	–	–	✓	–
23.	Female	61.2	✓	✓	✓	✓	–	✓	–	✓	✓	✓
24.	Body mass index	51.6	–	–	–	–	–	–	–	–	–	–
	Model selection frequency (%)		0.7	0.6	0.4	0.4	0.4	0.4	0.3	0.3	0.3	0.2

✓, variables included in the model; –, variables not selected.
* The number of variables in the model is given within parentheses.

Pooled parameter estimates of the best model for the dependent variable log(Audio-Visual Synesthesiae)

Predictors	Coefficient	Standard error	*P*-value		HPD CI low	HPD CI high	*n* missings	FMI	RIV
Drug dose	0.43	0.11	0.00	***	0.22	0.65	0	0.19	0.23
Positron emission tomography	-0.42	0.23	0.05		-0.88	0.01	0	0.53	1.07
Emotional Excitability (EWL)	0.27	0.14	0.04	*	0.01	0.54	224	0.39	0.62
Absorption (TAS)	0.51	0.15	0.00	***	0.22	0.81	312	0.52	1.02
Performance-Related Activity (EWL)	0.31	0.15	0.04	*	0.02	0.59	224	0.36	0.55
Hallucinogen-naïve	0.06	0.11	0.56		-0.15	0.28	40	0.15	0.18
General Inactivation (EWL)	-0.05	0.14	0.70		-0.32	0.21	224	0.39	0.61
Alcohol frequency	0.30	0.11	0.01	**	0.08	0.52	83	0.26	0.33
General Well-Being (EWL)	0.11	0.21	0.61		-0.30	0.52	224	0.49	0.90
Extroversion-Introversion (EWL)	-0.23	0.19	0.22		-0.62	0.14	224	0.45	0.78
Activity (ZKPQ)	0.05	0.12	0.66		-0.19	0.29	182	0.30	0.42
Neuroticism-Anxiety (ZKPQ)	-0.09	0.15	0.56		-0.38	0.20	182	0.41	0.66
Anxiety-Depressiveness (EWL)	0.10	0.14	0.50		-0.19	0.38	224	0.33	0.48
Impulsive Sensation Seeking (ZKPQ)	-0.12	0.16	0.49		-0.43	0.20	182	0.40	0.63
THC sometimes vs rarely	-0.13	0.12	0.25		-0.36	0.10	47	0.20	0.25
THC rarely vs never	-0.03	0.15	0.85		-0.31	0.26	47	0.14	0.17
Sociability (ZKPQ)	0.28	0.12	0.03	*	0.03	0.52	182	0.29	0.39
Aggression-Hostility (ZKPQ)	0.03	0.13	0.81		-0.23	0.28	182	0.35	0.51
Time of measuring	0.18	0.10	0.08		-0.02	0.38	0	0.08	0.09
Global Severity Index (SCL-90-R)	-0.16	0.13	0.25		-0.42	0.11	111	0.35	0.52
Female	-0.07	0.11	0.53		-0.28	0.15	0	0.20	0.24

Note. HPD = Highest Posterior Density. CI = Confidence Interval. FMI = Fraction of missing information. RIV = Relative increase in variance due to missingness.

Model selection for the dependent variable $\sqrt{Changed\ Meaning\ of\ Percepts}$

	Predictors	Step 1 (%)	Models selected in step 2 (the first 10 models are shown)*									
			1 (18)	2 (20)	3 (19)	4 (17)	5 (18)	6 (17)	7 (18)	8 (18)	9 (17)	10 (19)
1.	Drug dose	100.0	✓	✓	✓	✓	✓	✓	✓	✓	✓	✓
2.	Absorption (TAS)	99.8	✓	✓	✓	✓	✓	✓	✓	✓	✓	✓
3.	Performance-Related Activity (EWL)	99.8	✓	✓	✓	✓	✓	✓	✓	✓	✓	✓
4.	Hallucinogen-naïve	99.3	✓	✓	✓	✓	✓	✓	✓	✓	✓	✓
5.	Positron emission tomography	97.8	✓	✓	✓	✓	✓	✓	✓	✓	✓	✓
6.	General Inactivation (EWL)	94.5	✓	✓	✓	✓	✓	✓	✓	✓	✓	✓
7.	Emotional Excitability (EWL)	94.3	✓	✓	✓	✓	✓	✓	✓	✓	✓	✓
8.	Global Severity Index (SCL-90-R)	93.4	✓	✓	✓	✓	✓	✓	✓	✓	✓	✓
9.	General Well-Being (EWL)	92.7	✓	✓	✓	✓	✓	✓	✓	✓	✓	✓
10.	Extroversion-Introversion (EWL)	92.2	✓	✓	✓	✓	✓	✓	✓	✓	✓	✓
11.	Time of measuring	87.4	✓	✓	✓	✓	✓	✓	✓	✓	✓	✓
12.	Activity (ZKPQ)	85.8	✓	✓	✓	✓	✓	✓	✓	–	✓	✓
13.	Anxiety-Depressiveness (EWL)	85.8	✓	✓	✓	✓	✓	✓	✓	✓	✓	✓
14.	Sociability (ZKPQ)	84.3	✓	✓	✓	✓	✓	✓	✓	✓	–	✓
15.	Impulsive Sensation Seeking (ZKPQ)	83.8	✓	✓	✓	✓	✓	✓	✓	✓	✓	✓
16.	Alcohol frequency	82.0	✓	✓	✓	✓	–	✓	✓	✓	✓	✓
17.	Age	77.9	–	✓	✓	–	✓	–	✓	✓	–	–
18.	Aggression-Hostility (ZKPQ)	77.9	✓	✓	✓	✓	✓	–	✓	✓	✓	✓
19.	Neuroticism-Anxiety (ZKPQ)	74.5	✓	✓	✓	–	✓	✓	–	✓	✓	✓
20.	Years of education	64.5	–	✓	–	–	–	–	–	–	–	✓
21.	Female	63.2	–	–	–	–	–	–	–	–	–	–
22.	Daily smoker	59.2	–	–	–	–	–	–	–	–	–	–
23.	THC frequency	50.9	–	–	–	–	–	–	–	–	–	–
24.	Body mass index	49.5										
	Model selection frequency (%)		1.3	1.0	1.0	0.6	0.5	0.5	0.5	0.4	0.4	0.4

✓, variables included in the model; –, variables not selected.
* The number of variables in the model is given within parentheses.

Pooled parameter estimates of the best model for the dependent variable $\sqrt{Changed\ Meaning\ of\ Percepts}$

Predictors	Coefficient	Standard error	*P*-value		HPD CI low	HPD CI high	*n* missings	FMI	RIV
Drug dose	0.47	0.10	0.00	***	0.27	0.68	0	0.13	0.15
Absorption (TAS)	0.33	0.16	0.05	*	0.02	0.63	312	0.55	1.14
Performance-Related Activity (EWL)	0.56	0.16	0.00	***	0.26	0.87	224	0.47	0.85
Hallucinogen-naïve	0.24	0.11	0.02	*	0.03	0.46	40	0.15	0.18
Positron emission tomography	0.05	0.19	0.77		-0.32	0.42	0	0.32	0.45
General Inactivation (EWL)	0.19	0.14	0.17		-0.07	0.47	224	0.43	0.71
Emotional Excitability (EWL)	0.11	0.14	0.45		-0.16	0.38	224	0.45	0.80
Global Severity Index (SCL-90-R)	-0.03	0.13	0.81		-0.28	0.22	111	0.31	0.44
General Well-Being (EWL)	-0.13	0.22	0.58		-0.55	0.31	224	0.57	1.24
Extroversion-Introversion (EWL)	-0.09	0.17	0.63		-0.44	0.25	224	0.33	0.48
Time of measuring	-0.12	0.10	0.24		-0.31	0.08	0	0.10	0.11
Activity (ZKPQ)	-0.18	0.12	0.14		-0.42	0.06	182	0.35	0.52
Anxiety-Depressiveness (EWL)	0.07	0.14	0.63		-0.21	0.34	224	0.32	0.47
Sociability (ZKPQ)	0.03	0.13	0.81		-0.24	0.29	182	0.43	0.74
Impulsive Sensation Seeking (ZKPQ)	0.09	0.18	0.62		-0.27	0.42	182	0.52	1.03
Alcohol frequency	0.05	0.11	0.68		-0.16	0.26	83	0.22	0.28
Aggression-Hostility (ZKPQ)	0.09	0.12	0.50		-0.15	0.33	182	0.32	0.45
Neuroticism-Anxiety (ZKPQ)	-0.10	0.14	0.47		-0.38	0.17	182	0.35	0.52

Note. HPD = Highest Posterior Density. CI = Confidence Interval. FMI = Fraction of missing information. RIV = Relative increase in variance due to missingness.
